# Characterisation of the enzyme transport path between shipworms and their bacterial symbionts

**DOI:** 10.1186/s12915-021-01162-6

**Published:** 2021-11-01

**Authors:** Giovanna Pesante, Federico Sabbadin, Luisa Elias, Clare Steele-King, J. Reuben Shipway, Adam A. Dowle, Yi Li, Marta Busse-Wicher, Paul Dupree, Katrin Besser, Simon M. Cragg, Neil C. Bruce, Simon J. McQueen-Mason

**Affiliations:** 1 Centre for Novel Agricultural Products, Department of Biology, University of York, York, YO10 5DD UK; 2 Centre for Enzyme Innovation, School of Biological Sciences, Institute of Biological and Biomedical Sciences, University of Portsmouth, Portsmouth, PO1 2DY UK; 3 Bioscience Technology Facility, Department, of Biology, University of York, York, YO10 5DD UK; 4 Department of Biochemistry, University of Cambridge, Cambridge, CB2 1QW UK; 5 Institute of Marine Sciences Laboratories, Langstone Harbour, Ferry Road, Eastney, Portsmouth, PO4 9LY UK

**Keywords:** Shipworm, *Lyrodus pedicellatus*, Wood-borers, CAZymes, Crystalline style, Food groove, Lignocellulose, Bacteria, Symbiosis

## Abstract

**Background:**

Shipworms are marine xylophagus bivalve molluscs, which can live on a diet solely of wood due to their ability to produce plant cell wall-degrading enzymes. Bacterial carbohydrate-active enzymes (CAZymes), synthesised by endosymbionts living in specialised shipworm cells called bacteriocytes and located in the animal’s gills, play an important role in wood digestion in shipworms. However, the main site of lignocellulose digestion within these wood-boring molluscs, which contains both endogenous lignocellulolytic enzymes and prokaryotic enzymes, is the caecum, and the mechanism by which bacterial enzymes reach the distant caecum lumen has remained so far mysterious. Here, we provide a characterisation of the path through which bacterial CAZymes produced in the gills of the shipworm *Lyrodus pedicellatus* reach the distant caecum to contribute to the digestion of wood.

**Results:**

Through a combination of transcriptomics, proteomics, X-ray microtomography, electron microscopy studies and in vitro biochemical characterisation, we show that wood-digesting enzymes produced by symbiotic bacteria are localised not only in the gills, but also in the lumen of the food groove, a stream of mucus secreted by gill cells that carries food particles trapped by filter feeding to the mouth. Bacterial CAZymes are also present in the crystalline style and in the caecum of their shipworm host, suggesting a unique pathway by which enzymes involved in a symbiotic interaction are transported to their site of action. Finally, we characterise in vitro four new bacterial glycosyl hydrolases and a lytic polysaccharide monooxygenase identified in our transcriptomic and proteomic analyses as some of the major bacterial enzymes involved in this unusual biological system.

**Conclusion:**

Based on our data, we propose that bacteria and their enzymes are transported from the gills along the food groove to the shipworm’s mouth and digestive tract, where they aid in wood digestion.

**Supplementary Information:**

The online version contains supplementary material available at 10.1186/s12915-021-01162-6.

## Background

In nature, there are many organisms able to degrade lignocellulose, having evolved to utilise plant carbohydrates for their metabolism through the production of plant cell wall-degrading enzymes [1]. Bacteria and fungi are well known for their ability to digest lignocellulosic materials, and many terrestrial and marine invertebrates (including species of insects, nematodes, molluscs and crustaceans) are also able to produce endogenous cellulases. In most of these invertebrates, the digestion of woody biomass is made more efficient by the presence of bacterial or fungal symbionts, with only the isopods *Limnoria* ssp. and the amphipods *Chelura terebrans* being shown able to digest wood using endogenous enzymes alone [2–4]. Microbial symbionts playing these roles are normally found in the host in the digestive system, where the biomass is processed.

**Fig. 1:**
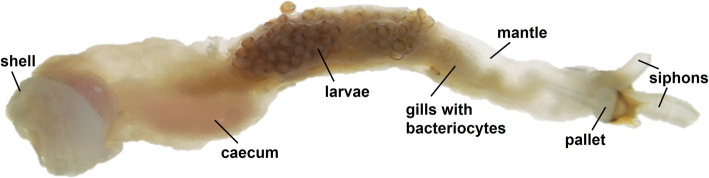
Shipworm anatomy. Light microscopic image of an adult specimen of the species *L. pedicellatus* showing its main organs

**Fig. 2:**
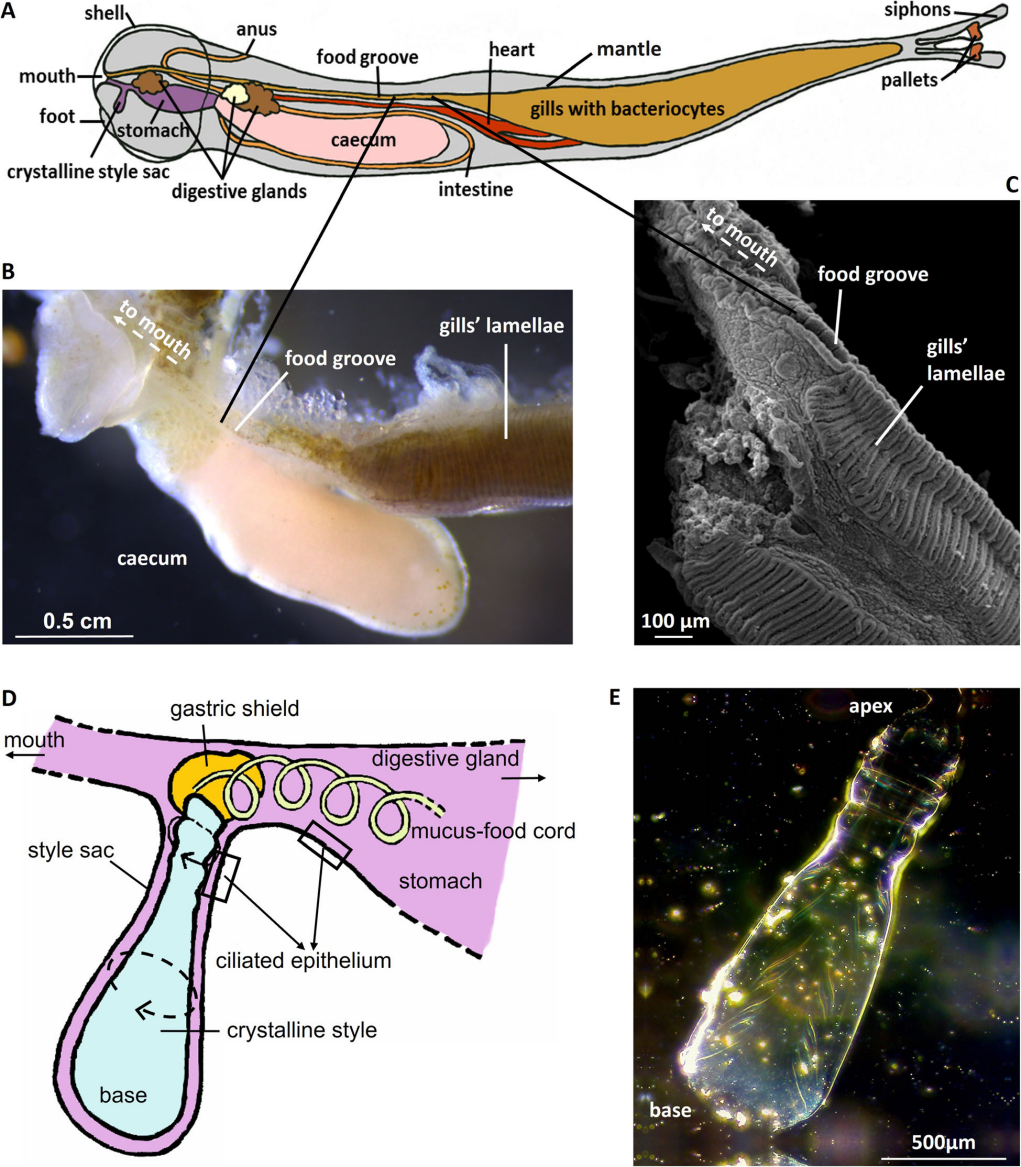
Food groove and crystalline style in *L. pedicellatus*. **A** Schematic view of *L. pedicellatus* and its main organs, including the food groove connecting the gills with the mouth. **B** Visible light microscopy of intact shipworm organs (after removal of the mantle), showing gills, food groove leading to the mouth and digestive caecum. **C** SEM image of *L. pedicellatus* gills. The single lamellae and the origin of the food groove tissue fold are clearly visible. **D** Diagram of the crystalline style region and its connection to the stomach. **E** Dark field microscopy image of a crystalline style (without crystalline style sac) of *L. pedicellatus*

Shipworms (Fig. 1) are marine xylophagous bivalve molluscs belonging to the Teredinidae family, which can live on a diet of wood [5, 6]. They use modified shells (Fig. 1) to burrow into the wood, which is ground into small particles that are ingested and digested [7]. Shipworms have an important role in the marine ecosystem, accounting for 70% of wood turnover in mangrove systems [8]. Shipworms are also considered pests, since their destructive action on man-made wooden structures such as piers, boats or navigation poles has substantial negative economic impacts [9, 10]. The ability of shipworms to feed on lignocellulose is dependent on the presence of endosymbiotic bacteria that live in the animal’s gills in specialised eukaryotic cells called bacteriocytes [11, 12]. These bacteria provide the animal with fixed nitrogen to supplement its diet [13–15], as well as hydrolytic enzymes to help wood digestion and secondary metabolites such as antibiotics [16–20]. Shipworms differ from other bivalve molluscs in possessing a caecum (Fig. 1 and Fig. 2A), packed with wood particles, which opens from the posterior stomach and in certain species occupies a considerable portion of the body cavity. The caecum is the main site of wood digestion and contains a large number of carbohydrate-active enzymes (CAZymes) of both endogenous and bacterial origin [21] despite harbouring few bacteria, belonging to ribotypes different from those found in the gills [22]. The prokaryotic fraction of CAZymes found in the caecum has been shown to be produced by bacteria resident in the spatially distant gills [23, 24], but until now the mechanism by which these bacterial enzymes end up in the lumen of the distant caecum has remained elusive. A duct connecting the gills to the oesophagus, located within the afferent branchial veins, has been reported in anatomical drawings of the shipworms *Bankia gouldi* and *Teredo furcifera* [25]. However, despite numerous later studies, no further evidence supporting the existence of this “duct of Deshayes” has been published [26], suggesting that a yet unidentified mechanism may be responsible for the translocation of enzymes from the gills to the caecum.

In filter feeding molluscs, food particles suspended in the water are captured by the action of cilia and mucus on the surface of the gills (Additional file 1: Fig. S1A, B). These food particles are then channelled into the food groove (Fig. 2A–C) in a stream of mucus propelled by the movement of surface cilia, which transports them to the animal’s mouth and digestive system [27]. In bivalves, an invagination of the stomach hosts a rotating gelatinous structure called the crystalline style (Fig. 2D, E), which is reported to accumulate digestive enzymes that are released by abrasion of the style against a chitinous area of the stomach wall, referred to as the gastric shield [28, 29]. The crystalline style has received little attention in shipworms, and its possible role in the interaction between host and symbionts has not been investigated.

In this paper, we present an in-depth investigation into the anatomical and molecular basis underpinning enzyme transport in shipworms and their bacterial endosymbionts. Based on data from meta-transcriptomic, meta-proteomic, X-ray micro-computed tomographic (micro-CT) and electron microscopy analyses, we propose that the food groove has been co-opted in shipworms to translocate bacteria and their secreted enzymes from the gills to the digestive system, and that the rotating crystalline style disrupts the incoming bacteria to further release their digestive enzymes, facilitating wood digestion. We also present in vitro biochemical characterisation of five prokaryotic lignocellulolytic enzymes identified in this complex symbiosis.

## Results

### Micro-CT analysis and scanning electron microscopy

**Fig. 3:**
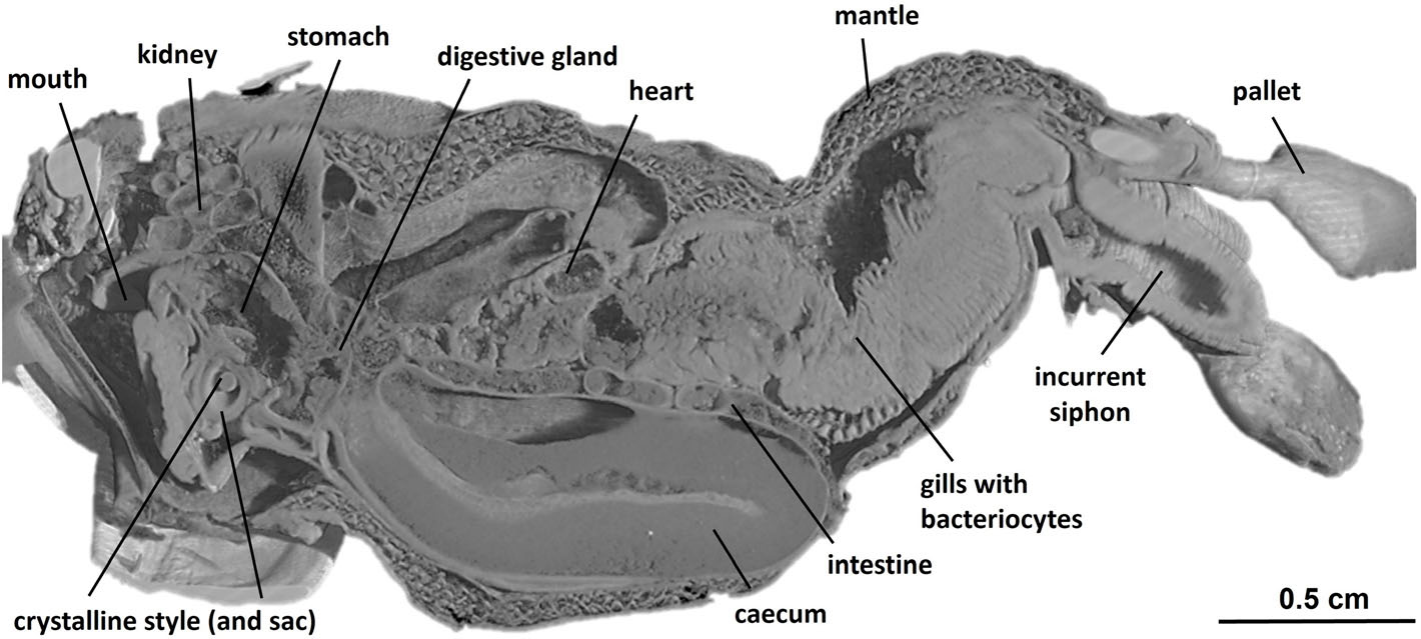
Micro-CT analysis of *L. pedicellatus*. A high-resolution three-dimensional rendering of an adult specimen, showing the anatomical arrangements in the mid-sagittal plane

In order to explore the internal anatomy of *L. pedicellatus*, a high-resolution three-dimensional rendering of an adult specimen was created using micro-CT (Fig. 3), of an animal extracted from the wood. This technique avoided disruption of the delicate anatomical structures, which inevitably occurs during manual dissection, and provided insight into the animal’s complex internal features. By analysing the 3D model generated, we were unable to detect a duct connecting the gills and digestive system via the route proposed by Sigerfoos [25]. Direct comparison with the illustrations of Sigerfoos was not possible due to significant modification of the gill structure in our specimen due to the presence of numerous brooded larvae. Previous descriptions of the duct were based on species that either do not brood larvae as part of their life cycle [25], or were not currently brooding at the time of study [30]. Future research could examine developmental stages prior to the onset of brooding. However, examination of sections from the anterior adductor to posterior adductor muscle, a region which encompasses the mouth, oesophagus and stomach, also failed to reveal any duct associated with the afferent branchial vein (Additional file 2: Fig. S2). Micro-CT analysis therefore reveals no evidence for a “duct” by which bacterial enzymes could move from the gills to the caecum. Further, scanning electron microscopy (Additional file 3: Fig. S3) shows how the food groove provides a direct connection between the gills and the digestive system in *L. pedicellatus*, and ciliated tracts leading from the crystalline style into the caecum have been mapped using coloured particles in live *L. pedicellatus* specimens [31].

### Bacterial CAZymes are found in the gills, caecum and food groove of *L. pedicellatus*

To identify the mechanism by which the bacterial CAZymes move from the gills to the digestive system, sections of the gill lamellae and food groove of *L. pedicellatus* were examined by TEM and SEM (transmission and scanning electron microscopy) in freshly harvested animals. These analyses showed eukaryotic cells (ranging from 10 to 50 μm and featuring organelles such as nuclei, mitochondria and Golgi apparatus) in the gill lamella tissues, which contained rod-shaped bacteria (Additional file 4: Fig. S4A-C), as previously described for bacteriocytes in other shipworm species [11]. These bacteria have the characteristic appearance of gram-negative species, with an inner and outer membrane separated by the periplasmic space (Additional file 4: Fig. S4C), as reported in other species of endosymbiont-bearing molluscs [32, 33].

**Fig. 4:**
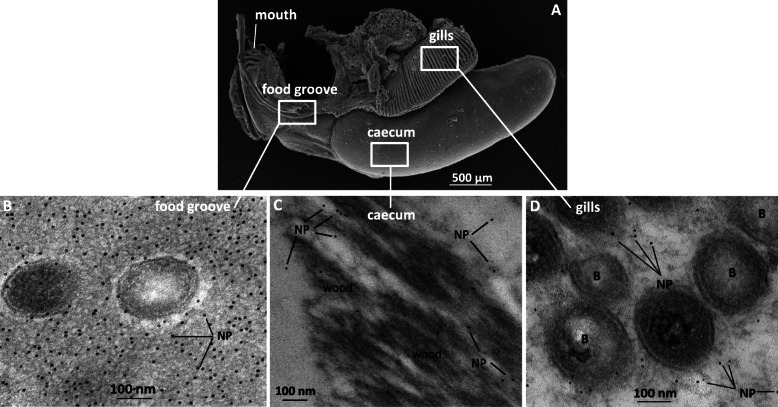
Immunogold labelling of *Lps*GH5_8 in *L. pedicellatus* using anti-protein serum. **A** SEM image illustrating the anatomical position of the gills, food groove, caecum and mouth in the shipworm body, from which the mantle has been removed. **B–D** Immunogold labelling of the food groove (**B**), caecum (**C**) and gills (**D**) performed with antibodies recognising the bacterial CAZyme *Lp*sGH5_8. Gold particles (small black dots) are observed in gill bacteriocytes (**D**), in the lumen of the food groove (**B**) and in the lumen of the caecum, typically on wood fragments (**C**). B = bacteria, NP = gold nanoparticle

To establish the route by which bacterial CAZymes produced in the gills reach the shipworm digestive system, immunogold labelling studies were performed (Fig. 4) using antibodies (Additional file 5: Fig. S5) raised against a bacterial glycoside hydrolase family 5 (GH5) identified as abundant both in our transcriptomic and proteomic analysis of shipworm organs (*Lp*sGH5_8, see next section on characterisation of bacterial CAZymes). TEM analysis of sectioned animals showed specific nanogold labelling in bacteriocytes in sections of the gill lamellae (Fig. 4D), and in the lumen of the food groove (halfway between gills and mouth, Fig. 4B), and in the lumen of the caecum (Fig. 4C), indicating the presence of the secreted protein. Nanogold particles in the food groove were found to be in close proximity to structures resembling, in size (~ 200 nm diameter, ~ 2 μm length), and features (inner and outer membrane, periplasmic space, granular content), the prokaryotic cells identified in the gills. In the caecum, the labelling was observed on and around the wood fragments (Fig. 4C), and no putative prokaryotic cells were visible. No labelling was observed on negative controls (pre-immune serum) (Fig. Additional file 6: Fig. S6).

### Bacterial CAZyme genes are transcribed in the gills and their proteins reach the crystalline style before entering the caecum

The crystalline style, found in bivalve molluscs and some gastropods, is an acellular rod rich in glycoproteins, located inside the crystalline style sac [34] (Fig. 2D,E). The crystalline style is thought to help digestion by mixing digestive enzymes, reducing the size of food particles and directing them to the appropriate compartment for digestion [35–44]. The proteins that form the structure of the style are thought to be produced by the style sac [45], although little is known about their identity and function. The possible role of the crystalline style in the symbiosis between gill bacteria and shipworms has not previously been investigated.

Here we carried out shotgun proteomics and transcriptome sequencing of the *L. pedicellatus* crystalline style and style sac respectively, and discovered that a significant portion of the proteome of the style (14.7%) is represented by CAZymes of both prokaryotic and eukaryotic origin (Table 1). Furthermore, analysis of relative transcript abundance in the gills, digestive glands and crystalline style sac revealed that all genes coding for eukaryotic CAZyme proteins found in the style are transcribed in the animal’s digestive glands, while the prokaryotic ones are exclusively transcribed in the gills (Table 2). Table S1 (Additional file 7) gives details of the annotation obtained with BlastX searches against the NCBI non-redundant database for the proteins detected in the crystalline style.

**Table 1:** Proteins of the crystalline style. Table showing the relative abundance (calculated from the emPAI score) of all the proteins identified through proteomic analysis of the crystalline style

Types of protein of the crystalline style	Relative abundance (%)
**Total of CAZy proteins**	**14,7**
Prokaryotic CAZy	8,00
Eukaryotic CAZy	6,70
**Total of non-CAZy proteins**	**85,3**
Mucin	22,49
Tubulin	15,03
Collagen	11,43
Apextrin-MACPF domain-containing	6,83
Hemocytin	4,10
Complement component	2,55
Fructose-1, 6-bisphosphate aldolase	2,05
Arginine kinase	1,59
Atrial natriuretic peptide-converting enzyme	1,21
Von willebrand factor	1,17
Perlucin	1,17
Dermatopontin	1,09
Glyceraldehyde-3-phosphate dehydrogenase	0,96
Matrilin	0,96
Oncoprotein-induced transcript	0,92
Leucine-rich repeat and death domain-containing	0,84
Actin	0,80
Triosephosphate isomerase	0,80
Niemann-Pick C1	0,75
Transaldolase	0,71
Beta-Ig-H3/fasciclin	0,63
Malate dehydrogenase	0,63
Aspartate aminotransferase	0,59
Tomoregulin-2-like	0,59
Periostin	0,54
Nerve hemoglobin	0,46
Phosphoenolpyruvate carboxykinase [GTP	0,42
Mitochondrial H+ATPase	0,38
CUB and EGF-like domain-containing	0,38
Heat shock protein	0,29
Uncharacterised	1,97
Others (<0.25)	0,97

**Table 2:**
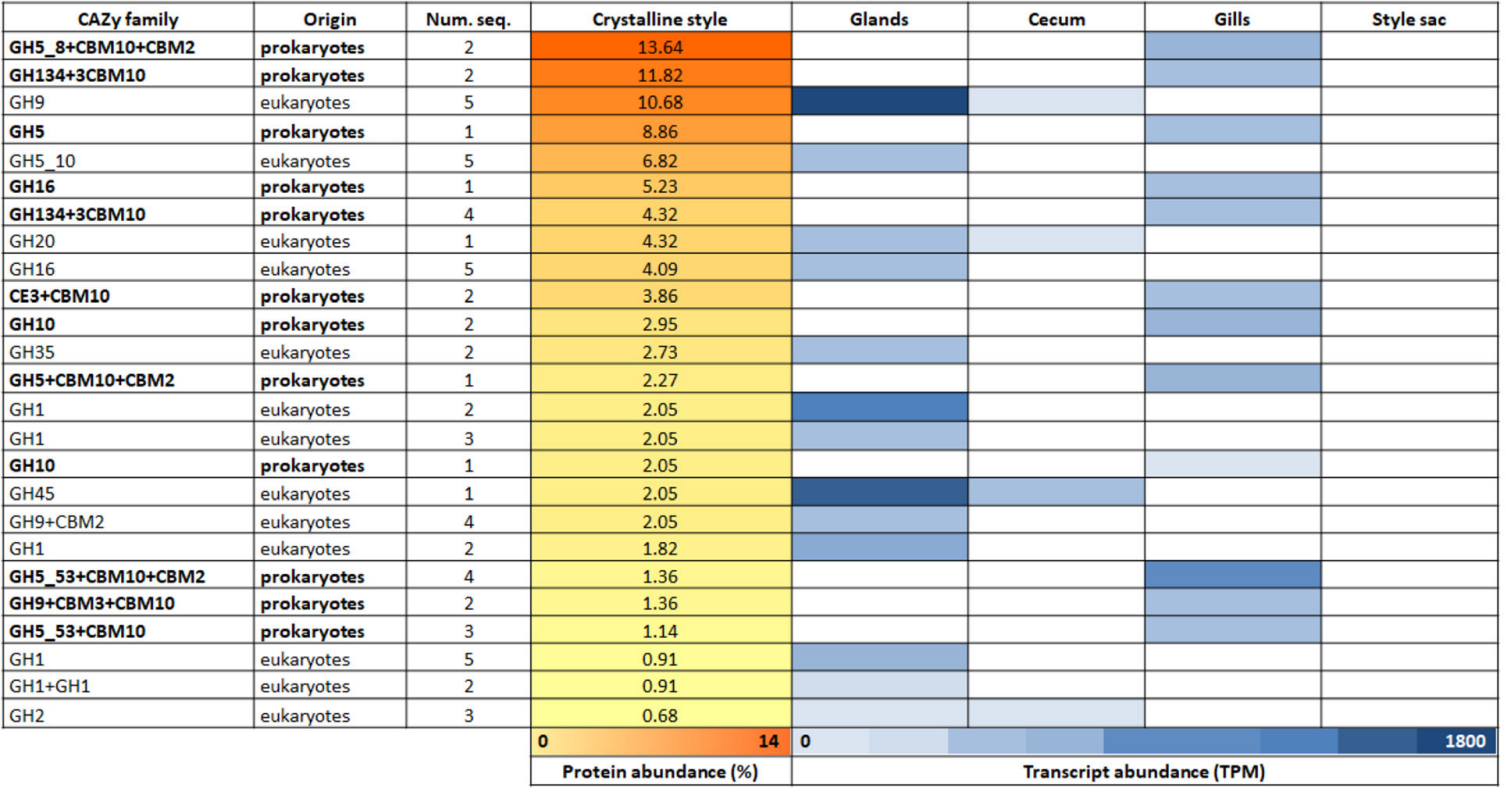
Proteomic and transcriptomic analysis of the crystalline style and its sac. Table listing the CAZymes identified in the crystalline style, with relative protein abundance (calculated from the emPAI score for the CAZymes only) illustrated with the yellow bars and relative transcript abundance (calculated as TPM for the best 10,000 transcripts) with blue cells. The prokaryotic proteins are shown in bold. Num. seq. indicates the number of significant matches

Bacterial CAZymes found in the crystalline style proteome are dominated by members of the GH5 (27.3% of all the CAZymes) and GH134 (16.1%) families, with a lesser contribution of proteins from the GH16 (5.2%), GH10 (5.0%) and carbohydrate esterase (CE) 3 families (3.9%). Most of the prokaryotic CAZymes found in the crystalline style possess carbohydrate binding modules (CBMs) belonging to family 10. The main substrates for which these CAZymes and associated modules have been reported to be active, are described in Table S2 (Additional file 8). The most abundant animal proteins identified by proteomic analysis in the crystalline style are structural proteins, mucin (22.5%), tubulin (15.0%) and collagen (11.4%). The main eukaryotic CAZy families present in the style are GH9 (12.7% of the CAZymes), GH7 (7.7%) and GH5 (6.8%), while GH20, GH16, GH35, GH45 and GH2 family proteins are found at 4.3%, 4.1%, 2.7%, 2.0% and 0.7%, respectively. Other abundant eukaryotic proteins in the crystalline style are annotated as complement component (2.5%), hemocytin-like proteins (4.1%) or containing a membrane attack complex/perforin (MACPF) domain (6.8%), all of which have a major role in innate and adaptive immunity [46–48].

In order to investigate whether the CAZymes found in the crystalline style are transported to the caecum and likely contribute to wood digestion, we repeated the proteomic analysis of the caecum contents of *L. pedicellatus* performed by Sabbadin and colleagues [21], using a more modern mass spectrometer, which allowed a greater dynamic range of detection and therefore a more complete identification of the proteome analysed. We found that all the CAZymes identified in the crystalline style are also present in the caecum (Additional file 9: Table S3). Only 17.3% of the CAZymes detected in the caecum are not found in the crystalline style and their coding genes are transcribed in the caecum itself, downstream of the crystalline style.

### Characterisation of CAZymes from gill bacterial symbionts

**Fig. 5:**
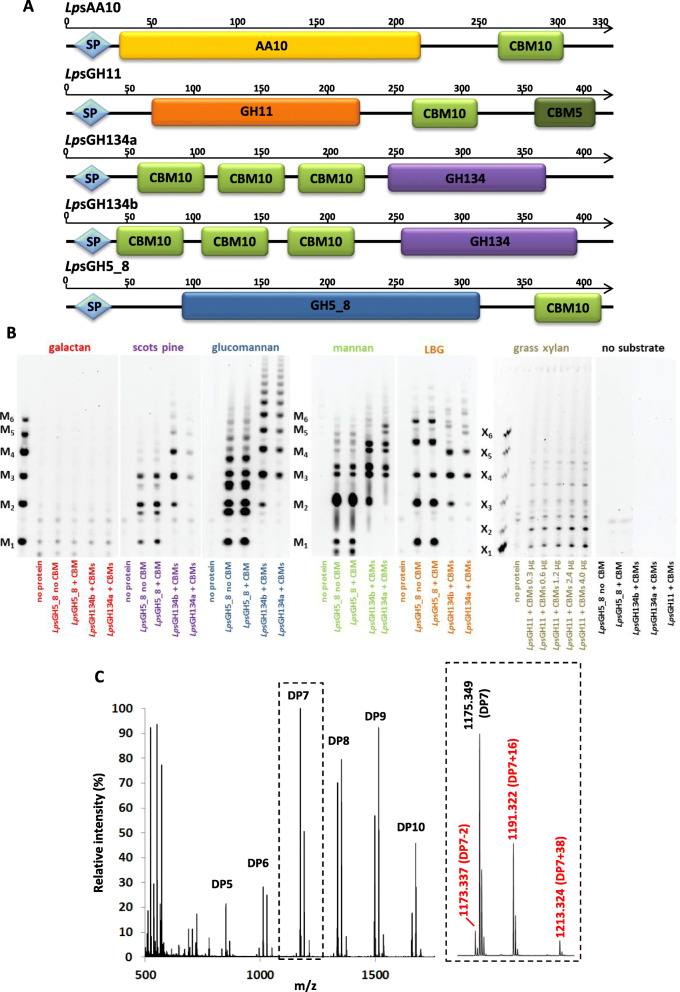
Characterisation of purified recombinant bacterial CAZymes encoded by the endosymbionts. **A** Schematic diagram showing the architecture of *Lps*GH5_8, *Lp*sGH11, *Lp*sGH134a, *Lp*sGH134b and *Lp*sAA10A, showing the N-terminal signal peptide for secretion and the main protein domains. **B** Polysaccharide analysis using carbohydrate gel electrophoresis (PACE) performed on different substrates (listed on top of the figure) for the bacterial proteins *Lps*GH5_8 (with and without its CBM), *Lps*GH134a, *Lps*GH134b and *Lps*GH11 with their CBMs. *Lps*GH11 was tested only on grass xylan (miscanthus stem AIR) and using different protein amounts (detailed in the picture), while the other enzymes were tested on a wider range of substrates using 1 μg of protein. LBG = locust bean gum. M1 to M6 are mannan standards containing from one to six mannosyl residues; X1 to X6 are xylan standards. The negative controls are those with no substrate (right panel) or no protein (first lane of each panel). **C** MALDI-TOF MS spectrum of products obtained after incubation of *Lp*sAA10A with PASC and gallic acid as electron donor. The main peaks correspond to mono- or di-sodiated adducts of C1-aldonic acids, imparting + 16 or + 38 m/z respectively, relative to the mono-sodiated unoxidised form. Smaller peaks for the mono-sodiated lactone (− 2) were also identified. All oxidised species are marked in red. The 100% relative intensity represents 1.0 × 10^4^ arbitrary units. Negative control reactions carried out with substrate only, substrate plus gallic acid and substrate plus *Lps*AA10A did not generate any oxidised products (see Additional file 13: Fig. S8). Inset shows the expanded mass spectra for DP7 (degree of polymerisation 7) products

The combined results from micro-CT analysis, electron microscopy, transcriptomics and proteomics indicate that bacterial CAZymes are transported by the food groove from the gills to the stomach, where they are found in the crystalline style together with eukaryotic CAZymes and are finally released into the caecum by ciliary currents. In order to define the activities of some of the main bacterial CAZymes found in *L. pedicellatus* proteome and transcriptome, recombinant versions were produced and characterised in vitro. Full-length coding sequences of five enzymes were successfully cloned from RNA extracted from bacteria located in the gills, heterologously expressed and purified. Selected targets belong to GH family 5 subfamily 8 (*Lp*sGH5_8, where *Lp*s stands for *Lyrodus pedicellatus* symbionts; accession LS999939; antibodies raised against this enzyme were used for all immunolabelling experiments described earlier), GH11 (*Lp*sGH11, accession LS999940), GH134 (*Lp*sGH134a and *Lp*sGH134b, accession LS999941 and LS999941 respectively) and Auxiliary Activity 10 (*Lp*sAA10A, accession LS999942). All native target proteins carried an N-terminal signal peptide for secretion, at least one CBM, and contained up to 10 disulphide bonds (Fig. 5A and Additional file 10 and 11: Table S4 and S5). BlastP searches against the NCBI non-redundant database (NCBI nr) found best matches with proteins from the gammaproteobacteria *Teredinibacter turnerae* or Alteromonadaceae Bs08 and Bs12 strains, two of the genetically distinct ribotypes previously identified within *Bankia setacea* endosymbiont populations [23].

The purified recombinant GH enzymes were tested in vitro on a range of polysaccharide substrates using carbohydrate gel electrophoresis (PACE) (Fig. 5B) and DNS-reducing sugar assays (Additional file 12: Fig. S7).

The assays revealed that *Lp*sGH5_8 (either with or without CBM) is active on a range of mannans, releasing mannose, mannobiose and longer mannooligosaccharides from glucomannan, locust bean gum (LBG), mannan and Scots pine wood.

Consistent with its predicted function as a β-1,4-xylanase, *Lps*GH11 expressed with both its CBMs showed activity on arabinoxylan and xylan. PACE analysis of the products released from grass xylan (miscanthus stem alcohol-insoluble residues) indicated the release of xylose, xylobiose and decorated xylooligosaccharides.

*Lp*sGH134a and *Lp*sGH134b, which are putative endo-β-1,4-mannanases based on similarity to known CAZymes, were mainly active on glucomannan, mannan, LBG and galactan. PACE analysis showed the absence of mannose monosaccharides (and disaccharides for *Lp*sGH134a), suggesting that these bacterial hydrolases act as endo-mannanases requiring mannosyl residues at the − 2, − 1, + 1 and + 2 subsites of the enzyme.

Activity assays with purified, copper-bound *Lp*sAA10A [lytic polysaccharide monooxygenases (LPMO) domain only] were carried out on a panel of cellulosic, hemicellulosic and chitinous substrates (Methods) in the presence of the electron donor gallic acid. Samples were analysed by MALDI-TOF MS and peak masses of the reaction products compared to previously published data [49, 50]. This revealed a predominant C1-oxidation pattern and generation of C1-aldonic acids on both phosphoric acid swollen cellulose (PASC, Fig. 5C) and microcrystalline cellulose (Avicel, Additional file 8: Fig. S8D) by *Lp*sAA10A in presence of an external electron donor. Oxidised products were not detected in the negative controls (Additional file 13: Fig. S8A-C and E-G).

## Discussion

We used a range of microscopic, transcriptomic, proteomic and biochemical analyses to shed light on the anatomical adaptations and the dynamics of digestive enzyme distribution in *Lyrodus pedicellatu*s. We have confirmed that the genes for bacterial enzymes found in the digestive caecum are transcribed in the gills [23], and our microscopy and micro-CT analyses indicate that the food groove is the most likely direct connection between the gills and the mouth (which leads into the caecum). *Lp*sGH5_8 was detected in the crystalline style through proteomics analysis, and localised in the gills, as well as in the lumen of the food groove and of the caecum, using immunogold labelling. The density of nanogold particles found in the food groove lumen was much higher than in the gill bacteriocytes, suggesting that bacterial secretion of CAZymes mostly occurs after the bacteria have left the gills. Interestingly, the immunogold signal in the food groove was mostly localised around bacterial cells, indicating that both free bacteria and their secreted enzymes may be translocated via the food groove. Finally, the presence of nanogold particles, but not bacteria-like structures, in the lumen of the caecum suggested that bacterial cells are likely disrupted upon leaving the food groove, either in the mouth or within the stomach.

Our proteomics analysis also showed that eukaryotic and prokaryotic CAZymes account for 15% of all the proteins in the crystalline style, a rotating rod-shaped structure found in the animal’s stomach. The apparent accumulation of bacterial CAZymes in the shipworm style, and the lack of visible bacterial cells downstream of it, suggests that this rotating structure is responsible for disrupting incoming bacteria (as well as food particles) from the food groove, potentially releasing more wood digestive enzymes. Our analysis of the style found a highly abundant protein belonging to the Membrane Attack Complex/Perforin (MACPF) perforin superfamily, involved in punching holes in the membranes of Gram-negative bacteria as part of the animal immune system [47]. The possible role of the style as a bacterial “grinder” upstream of the caecum is also indirectly supported by previous FISH investigations that showed only very sparse bacterial populations in the caecum [22].

**Fig. 6:**
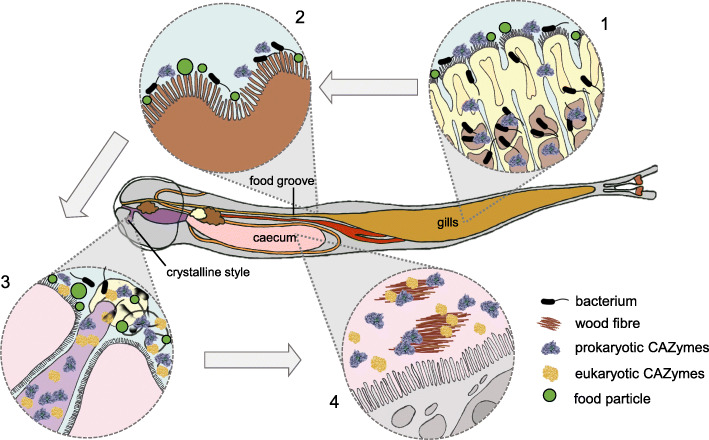
Diagram showing the shipworm anatomy and the proposed route by which bacteria and their secreted enzymes are transported. (1) Bacteria and their secreted CAZymes are found in the gill’s lamellae and in the ciliated epithelium, together with food particles. (2) Bacteria, prokaryotic CAZymes and food particles are channelled into the food groove and propelled by the movement of surface cilia towards the mouth and stomach. (3) In the stomach, the crystalline style grinds the incoming food particles and bacteria against the gastric shield, effectively releasing more CAZymes and mixing them with endogenous enzymes coming from the animal’s digestive glands. (4) A mix of eukaryotic and prokaryotic CAZymes reaches the caecum, where they digest the wood fibres

Based on our observations and available literature, we suggest the following scenario (Fig. 6). Bacterial endosymbionts residing in the gill bacteriocytes are either passively or actively expelled from the gill’s tissue and incorporated in the mucous stream of food particles originated from filter feeding. Bacteria (together with food particles) are channelled into the food groove, propelled by the movement of surface cilia and they start releasing abundant CAZymes, possibly as a result of cell death induced by the host’s immune system. Through the food groove, bacteria are transported to the animal’s mouth and stomach, where the rotating crystalline style, enriched with bacteria-lysing MACPF perforin, grinds the incoming food particles, wood fragments and bacteria against the gastric shield, effectively releasing more CAZymes and mixing them with endogenous enzymes coming from the animal’s glands. The homogenised mixture of enzymes and food is transported by tracts of cilia [31] from the region of the style to the caecum, where the combined action of bacterial and eukaryotic CAZymes degrades lignocellulose and releases simple sugars that are taken up by the abundant glucose transporters (solute carrier family 2 transporters and sodium-dependent glucose transporters) previously identified in the caecum tissues [21].

In the present work, we have also characterised some of the major bacterial CAZymes identified in the gills, food groove and crystalline style of *L. pedicellatus* (belonging to GH families 5, 134 and 11, and the AA10 family), and identified activities on cellulose and hemicelluloses, particularly galacto-glucomannan and xylans. Our microscopy and shotgun proteomics studies have shown that GH5_8, a highly active galacto- and glucomannanase, is abundant both in the food groove and in the crystalline style. Strong hemicellulolytic activities are indeed to be expected in animals grown in blocks of Scots pine, a gymnosperm wood particularly rich in mannans [51]. The results are in line with the findings of O’Connor and colleagues [23], who identified numerous mannanases, mannosidases and xylanases in the caecum contents of the shipworm *B. setacea*. We also managed to obtain the recombinant version of a bacterial AA10 lytic polysaccharide monooxygenase (LPMO) from *L. pedicellatus* and characterised its ability to cleave crystalline and amorphous cellulose at the C1 position, similarly to a previously characterised bacterial AA10 from *Teredinibacter turnerae* [52]. Our combined omics analysis suggests that, although *L. pedicellatus* carries endogenous AA15 LPMO sequences, both their gene expression and protein abundance are very low in the digestive system (digestive glands, crystalline style, caecum) and that bacterial LPMOs have been co-opted towards wood digestion. This strategy is in striking contrast with the solution evolved by the firebrat *Thermobia domestica*, a terrestrial insect that exploits a complex array of endogenous LPMOs to digest cellulose [21].

Our work provides new insight into the evolution of complex symbioses between animals and intracellular bacteria. Although intracellular symbionts involved in supplementing nutrients to host are common among insects such as aphids and weevils [53, 54] and marine invertebrates such as vestimentiferan tubeworms and bivalve molluscs [55, 56], shipworms offer the most striking example of symbionts that are resident in organs spatially distant from the site of food digestion and nutrient uptake. This mechanism allows shipworms to take full advantage of the abundant carbohydrates found in wood without competition from symbiotic bacteria; however, it creates the logistical problem of transporting necessary nutrients and enzymes to a distant organ. Similar arrangements are rare in nature. One example is the tortoise beetle *Cassidia rubiginosa*, which hosts pectin-degrading bacteria within special organs of the reproductive tract [57]. Shipworms have elegantly solved the problem by adopting a transport system that was already in place as part of their filter feeding behaviour. This evolutionary stratagem enabled them to fill an ecological niche in the marine ecosystem that was only partially exploited by other organisms. Our observation that bacteria appear to be transported along the food groove towards the digestive system in *L. pedicellatus* suggests that shipworms could be using this organ not only to relocate bacterial CAZymes, but also to recycle bacteria as a source of nutrients, particularly nitrogen, which is scarce in wood. Indeed, marine bivalves have been shown to feed on bacteria as a food source when phytoplankton is in short supply [58, 59]. It is possible that the use of the food groove as a transport system for bacterial products is widespread among bivalves and other molluscs, where endosymbiotic bacteria are commonly hosted in the gills [56]. Recycling bacterial products by transporting them via the food groove could supplement their nutrition and allow them to thrive in extreme environments, such as deep marine hydrothermal vent ecosystems [60].

## Conclusions

We have identified a novel putative mechanism of translocation of bacterial enzymes across distant organs in shipworms, and provided evidence that the food groove and the crystalline style may play key roles in it. This mechanism originates from a unique combination of anatomical and molecular features evolved by shipworms for the difficult task of digesting wood lignocellulose.

## Methods

### Animal rearing and dissection

Shipworms of the species *Lyrodus pedicellatus* from the Atlantic lineage [61] were used for our work. Samples were collected from the pier of Portsmouth Harbour (50° 47′ 47″ N, 1° 01′48″ W). Larvae from the original wood were used to infest logs of Scots pine, which were kept in tanks in the laboratories of the Institute of Marine Science, University of Portsmouth. To rear the animals, the water was taken directly from the Langstone Harbour (34 PSU salinity) and kept aerated and at a temperature of 15–18 °C using a flow-through system. The wood logs were opened by splitting them with a hammer and screwdriver, and the animals were then extracted with tweezers, placed in sea water containing EDTA-free protease inhibitors (1% v/v, Thermo Scientific) and kept on ice until dissection to anesthetise them. Species identification was performed using the pallets as described in [62]. The dissections were performed using a stereomicroscope (Leica MZ6) after removing the mantle to expose the organs.

### Microscopy

#### Scanning and transmission electron microscopy

Ten shipworms ranging in size from 4 to 8 cm were freshly extracted for all the microscopy experiments. For transmission electron microscopy, caecum, gills and food groove samples were fixed for 1–2 h at room temperature in primary fixative (4% formaldehyde (w/v), 2.5% (w/v) glutaraldehyde in 100 mM sodium phosphate buffer pH 7.2), and then washed (3 × 10 min) in 100 mM sodium phosphate buffer pH 7.2. Samples were then incubated in secondary fixative (1% osmium tetroxide in 100 mM sodium phosphate buffer pH 7.2) for 1 h on ice and dehydrated through a graded ethanol series (15 min each), followed by two washes (5 min each) in epoxy propane. Samples were infiltrated with a series of epoxy propane/Epon araldite (25%, 50%, 75% Epon Araldite with a minimum of 1 h at each stage, all at 30 °C) plus at least two changes of Epon araldite resin over 24 h at 30 °C, and polymerised at 60 °C for 48 h in flat embedding moulds. Pale gold (70–90 nm) ultra-thin sections were cut with a Diatome diamond knife using a Leica Ultracut UCT microtome and mounted on hexagonal 200-mesh nickel grids. Sections were post-stained with 2% (w/v) aqueous uranyl acetate (10 min) followed by lead citrate (5 min) in a carbon dioxide-free chamber and viewed using a FEI Tecnai 12 BioTWIN G2 TEM operating at 120 kV. Images were captured using AnalySIS software and a Megaview III CCD camera.

For scanning electron microscopy, the samples were fixed in 4% (v/v) glutaraldehyde in a cacodylate buffer (0.2 M sodium cacodylate, 0.3 M sodium chloride, 2 mM calcium chloride) for 2 h at room temperature and then rinsed once in buffer for 30 min. Samples were then taken through an ethanol dehydration series (50-70-100% ethanol and twice in 100% acetone, each stage for 30 min), critical point dried and then mounted on aluminium stubs using adhesive carbon tabs. Sputter coating was carried out under an argon atmosphere using a gold and palladium target, at a voltage of 1.4 kV using a current of approximately 18 mA for 3 min. Specimens were examined using a Zeiss MA10 Scanning Electron Microscope with an accelerating voltage of 20 kV and the Zeiss Smart software.

#### Immunogold labelling

Embedding for immunolabeling proved difficult and several attempts were made to allow resin infiltration and at the same time preserve antigenicity. Freshly dissected shipworm tissues (food groove, gills and caecum) were fixed with 4% paraformaldehyde, 0.2% glutaraldehyde in sodium cacodylate buffer (0.2 M sodium cacodylate, 0.3 M sodium chloride, 2 mM calcium chloride, pH 7.4) on ice in a vacuum chamber for 2 h, then on a rotator without vacuum for a further 12 h at 4 °C. Samples were washed in 0.2 M sodium cacodylate buffer (three washes of 20 min each) and dehydrated through a graded ethanol series initially on ice (50%) and subsequently at − 20 °C on a rotator (70%, 90%, 100%) with 20 min at each stage and two changes of 100% ethanol. Ethanol was gradually replaced with LR Gold resin (1:2, 1:1, 2:1 resin:ethanol) with 1 h at each stage, followed by three changes of 100% LR Gold resin, 12 h each, all at − 20 °C on a rotator. Tissues were embedded in closed gelatine capsules and polymerised with UV light at − 20 °C for 24 h, followed by 24 h at − 10 °C. Pale gold (70–80 nm) ultra-thin sections were cut with a Diatome diamond knife, using a Leica Ultracut UCT microtome, and mounted on hexagonal 200-mesh nickel grids. All immunolabeling steps were achieved by floating grids on droplets of reagent. Sections were incubated in blocker (3% BSA in phosphate-buffered saline—PBS, 137 mM NaCl, 2.7 mM KCl, 10 mM Na_2_HPO_4_ and 1.8 mM KH_2_PO_4,_ pH 7.0) for 30 min at ambient temperature before incubation with primary antibodies against the bacterial *Lp*sGH5_8 (see further down for details on antibodies production) diluted 1:100 in 1% BSA in PBS at 30 °C for 1 h, followed by washing with PBS at ambient temperature (three brief washes followed by three washes of 10 min each). Sections were incubated in secondary antibody (goat anti-rabbit IgG conjugated to 10 nm gold) diluted 1:100 in 1% BSA in PBS for 1 h at 30 °C, followed by washes with PBS as before, and subsequently with ultrapure water. All immunolabeling procedures included negative controls treated exactly as the samples: both pre-immune controls (diluted 1:100 in 1% BSA in PBS) and buffer only (1% BSA in PBS). Sections were post-stained with 2% (w/v) aqueous uranyl acetate (10 min), then lead citrate (5 min) in a carbon dioxide-free chamber and viewed using a FEI Tecnai 12 BioTWIN G2 operating at 120 kV. Images were captured using AnalySIS software and a Megaview III CCD camera.

#### Antibody production and purification

Two milligrams of purified recombinant bacterial *Lp*sGH5_8 (see further sections for cloning, expression and purification) were used to raise polyclonal antibodies in rabbits (ProteoGenix, France). To enrich the serum for antigen-specific antibodies, affinity columns were made using recombinant *Lp*sGH5_8. Recombinant protein preparations were dialysed against coupling buffer (0.1 M NaHCO_3_, 0.5 M NaCl, pH 8.3) and bound to CNBr-activated Sepharose™ 4 Fast Flow resin (GE Healthcare Life Sciences) followed by affinity purification of an aliquot of the crude antibody serum according to the resin manufacturer’s instructions. The pre-immune serum was subject to the same purification procedure. Purified antibody and pre-immune serum fractions were characterised for their affinity by western blotting using both recombinant *Lp*sGH5_8 and *L. pedicellatus* caecum fluids (Additional file 5: Fig. S5). Fractions showing the highest titre and no unspecific binding were selected for immunogold labelling.

### Micro-CT

#### Sample preparation

One adult *Lyrodus pedicellatus* (Quatrefages, 1849) specimen, measuring 3.6 cm in length and 1.62 cm in width, was used for micro-CT scanning. The specimen was reared at the Institute of Marine Sciences, University of Portsmouth, UK, and extracted from wood in 2012. The sample was fixed in 4 % v/v glutaraldehyde in a cacodylate buffer (0.2 M sodium cacodylate, 0.3 M sodium chloride, 2 mM calcium chloride) for 1 h at room temperature, rinsed three times in buffer for 10 min each, post-fixed in 1 % w/v aqueous osmium tetroxide for 1 h and rinsed three times in seawater for 10 min each. Samples were then immediately ethanol dehydrated and dried with hexamethyldisilazane (HMDS).

#### Micro-computed tomography

The specimen was mounted onto the sample holder and secured using glue, and scanned at the Ghent University Centre for X-ray Tomography (UGCT), Woodlab-UGent, using a scanner developed at UGCT. The scanner consisted of two X-ray tubes and two X-ray detectors, specifically designed to obtain very high-resolution scans as well as scans of larger objects. Scans were carried out using a microfocus X-ray tube in combination with a Varian flat-panel detector with an exposure time of 1500 ms, a rotation angle of 0.25° resulting in an average scan time of 45–60 min and an approximate voxel pitch of 2.5 μm. Details of the scanner are outlined in Masschaele et al. [63] and Van den Bulcke et al. [64, 65]. Due to large specimen size, two stacked scans were performed. The dataset was reconstructed using the Octopus software package with beam hardening correction. The two reconstructed volumes were then loaded in VGStudio MAX and stitched into a single stack of cross-sections. All resulting image and video analysis was performed using visualisation software myVGL.

### Transcriptomics

#### RNA extraction and sequencing

Gills, digestive glands and caecum from three healthy adult *L. pedicellatus* were dissected and prepared for the paper published by Sabbadin and colleagues [21], using Ribosomal RNA depletion with a RiboZero™ Magnetic Gold Kit (Epidemiology) (Epicentre) in order to isolate both eukaryotic and prokaryotic mRNA. RNA-Seq libraries were prepared from each mRNA sample according to the Ion Total RNA-Seq kit v2 (Thermo Fisher Scientific). Templates were synthesised from mRNA libraries using the Ion OneTouch 200 Template Kit v2 DL on a OneTouch system (Thermo Fisher Scientific) and sequenced on an Ion Torrent PGM™ using a 318 chip (IonPGM200Kit; Thermo Fisher Scientific). All raw sequence data are available in NCBI under BioProject PRJNA412369 (SRA files: SRR6106265, SRR6106266, SRR6106267, SRR6106268, SRR6106269, SRR6106270, SRR6106271, SRR6106272, SRR6106273).

Crystalline style sacs were freshly dissected from 38 animals (which were then pooled together), flash frozen in liquid nitrogen and stored at − 80 °C. Total RNA was extracted using TRIzol® Reagent (Thermo Fisher Scientific), DNase treatment was carried out with Turbo DNA-free (Ambion), RNA was cleaned with RNA Clean & Concentrator™-5 (Zymo Research) and then quantified with a Qubit 3.0 Fluorometer and Agilent TapeStation. RNA depletion for both eukaryotic and prokaryotic ribosomal RNA was performed with the Ribo-Zero^TM^ Magnetic Gold Kit Epidemiology (Epicentre) and mRNA was then concentrated using RNA Clean & Concentrator™-5 (Zymo Research). The sequencing of the crystalline style sacs was performed at the Next Generation Sequencing Facility at the University of Leeds with HiSeq3000 using Illumina Technology to generate the required 150 bp paired end data. After rRNA depletion, library construction was completed using Illumina’s TruSeq stranded mRNA library protocol, starting at the RNA fragmentation step as suggested by Illumina.

#### Transcriptome assembly and analysis

Two meta-transcriptomes were assembled, using raw EST sequencing reads from digestive gland, caecum and gills (meta-transcriptome 1) [21] and from crystalline style sacs (meta-transcriptome 2), respectively. The raw EST sequencing reads were trimmed using Trimmmatic (part of the Galaxy tool [66, 67]) and were assembled into contigs using the Trinity software v. 2.8.3 [68].. Raw reads were mapped back onto the contigs and gene expression levels were calculated as TPM values (transcripts per kilobase million) using Salmon as part of the online tool Galaxy [66, 69] using standard parameters. Annotation of the contigs was performed by BlastX searches against the non-redundant database of the NCBI. The online software dbCAN (DataBase for automated Carbohydrate-active enzyme ANnotation) [70] was used to search for carbohydrate-active domains. Results with an e-value < 1e^−10^ and those with a CBM (Carbohydrate Binging Module) but no annotation were excluded, as well as CAZymes (Carbohydrate-Active enZYmes) belonging to the class of glycosyl transferases.

### Proteomics

The following protocol describes the preparation and analysis of caecum and crystalline style samples. The caeca of five animals grown on Scots pine were dissected in 50 mM sodium phosphate buffer pH 7 and the content (food particles and enzymes) was isolated and pooled together. Similarly, 21 crystalline styles were dissected and washed three times in PBS buffer. Caecum and crystalline styles samples were then boiled for 10 min in denaturing buffer (1% SDS, 2.5% beta-mercapto ethanol, 175 mM DTT), centrifuged, and the supernatant was run into a 10% polyacrylamide gel. The protein bands were excised and digested with trypsin, and the resulting peptides were analysed by label-free LC-MS/MS. Tandem mass spectra were searched against the combined meta-transcriptomes (digestive gland, caecum, gills and crystalline style sac) of *L. pedicellatus* (which includes both eukaryotic and prokaryotic sequences) using the Mascot search programme. emPAI values were converted into molar percentages, the identified proteins were ranked based on relative abundance and annotated using BlastX versus non-redundant NCBI databases. CAZy annotation was carried out using the online tool dbCAN [70].

CAZy annotation was carried out using the online software dbCAN and results with an e-value < 1e^−10^, with a CBM but no CAZy module, and CAZymes belonging to the class of glycosyl transferases were excluded. Signal peptides were identified using the server SignalP 4.1 for either eukaryotes or gram-negative bacteria (www.cbs.dtu.dk/services/SignalP/).

### Bacterial CAZymes cloning, expression, purification and biochemical characterisation

RNA was extracted from the gill tissue using the TRIzol® method (Thermo Fisher Scientific), cleaned with RNA Clean & Concentrator™-5 (Zymo Research) and a polyA tail was added using the poly(A) polymerase and protocol from Takara [71]. cDNA was produced using the SuperScript® II reverse transcriptase (Thermo Fisher Scientific) with a oligo-dT primer and was purified with the Clontech NucleoSpin PCR Clean-up and gel extraction Kit (Clontech). The DNA sequences encoding the bacterial proteins *Lp*sGH5_8, *Lp*sGH11, *Lp*sGH134a and *Lp*sGH134b were amplified from cDNA without their signal peptide using the primers and PCR setting listed in Table S6 (Additional file 14) and they were cloned with the StrataClone Blunt PCR Cloning Kit (Stratagene); the sequences were verified by Sanger sequencing. *Lp*s*AA10A* was not successfully cloned from the cDNA and therefore a synthetic version of the gene was codon optimised for *E. coli* expression by GeneArt.

The In-Fusion HD cloning kit (Takara) was used for cloning *Lp*s*GH5_8*, *Lp*s*GH134a* and *Lp*s*GH134b* into the vector pET52b+, which has N-terminal Strep-Tag II followed by the human rhinovirus (HRV) 3C protease cleavage site, and a C-terminal His-Tag. The vectors were then transformed into *E. coli* Rosetta-Gami^TM^2(DE3) competent cells by heat shock.

*Lp*s*GH11* was cloned into the vector pOPINS3C [72], which contains an N-terminal His-Tag, followed by a Halo-Tag for improved soluble expression and the HRV 3C protease cleavage site, and no signal peptide for secretion. The vector was transformed into *Spodoptera frugiperda* 9 (Sf9) insect cells by heat shock.

*Lp*s*AA10A* was cloned, without its CBM, using the In-Fusion HD cloning kit (Takara) into a modified pET26 vector containing at the N-terminus the pelB leader sequence to direct protein production to the periplasm, and a C-terminal Strep-tag. The construct was transformed into Rosetta^TM^2(DE3) competent cells by heat shock.

#### Expression and purification

The *E. coli* bacterial cells containing *Lp*sGH5_8, *Lp*sGH134a and *Lp*sGH134b constructs were grown in LB broth supplemented with carbenicillin (50 μg/ml) and chloramphenicol (34 μg/ml) at 37 °C until OD_600_ = 0.7 and then induced with isopropyl β-D-1-thiogalactopyranoside (IPTG) 1 mM and grown overnight at 20 °C and 200 rpm. After harvesting the cells were pelleted, suspended in phosphate-buffered saline (PBS) with 0.01 mM 4-(2-aminoethyl) benzenesulfonyl fluoride hydrochloride (AEBSF) and were lysed by sonication. After addition of 5 mM MgCl_2_ and DNaseI (0.025 U/μl) and filtering through a 0.45-μm filter, the supernatant was run through a 5-ml StrepTrap column, washed with PBS and eluted with 2.5 mM desthiobiotin in PBS. The eluted fractions containing absorbance peaks were analysed by SDS/PAGE to confirm the presence of the recombinant protein, combined together and the strep-tag was removed with the HRV Turbo protease (ABnova) at a ratio of 1:100 overnight at 4 °C and gentle shaking. After removal of the HRV, gel filtration was performed with a HiLoad 16/600 Superdex 75 pg column (Ge Healthcare) and the relevant peaks were verified by SDS/PAGE.

Expression of *Lp*sGH11 in the Sf9 insect cells was performed using the baculovirus expression system [73] with a virus dilution of 1:1000. Once harvested, the cells were pelleted, resuspended in the lysis buffer I-PER insect cell protein extraction reagent (Thermo Fisher Scientific) with 5 mM MgCl_2_ and DNaseI (0.025 U/μl) and incubated on ice for 10 min. They were then pelleted and the supernatant was affinity purified with a pre-equilibrated HisTrap 5-ml column. Ni affinity chromatography was run with an elution gradient of 30 to 500 mM imidazole. Fractions were collected and analysed by SDS PAGE. Fractions containing the protein were pooled and concentrated to 2.5 ml and were run on a HiLoad 16/600 Superdex 75 pg column (Ge Healthcare) and the relevant peaks were verified by SDS/PAGE. The fractions containing the protein were concentrated to 1 ml and the tags were removed with the HRV Turbo protease (ABnova) at a ratio of 1:100 overnight at 4 °C and gentle shaking. Purification was carried out manually using a 5-ml HisTrap column and a gradient of 20–500 mM imidazole. The fractions were run by SDS-PAGE to confirm tag cleavage.

*E. coli* bacterial cells expressing the *Lp*sAA10A were grown in M9 Minimal Medium, containing 1% (w/v) glucose and the appropriate antibiotics at 37 °C until OD_600_ = 0.7. The culture was induced with IPTG (0.1 mM) and grown overnight at 20 °C. Cells were harvested by centrifugation, resuspended in 50 ml of 50 mM Tris-HCl/20% sucrose (pH 8.0) for each litre of original culture and kept on ice for 30 min. After centrifugation at 8000 rpm for 10 min the supernatant was discarded, the cells were resuspended in 50 ml of 5 mM MgSO_4_ for each litre of original culture and kept on ice for 30 min. After centrifugation the supernatant, containing the periplasmic fraction, was equilibrated with 0.2 M Na phosphate buffer pH 7.6 to a final concentration of 50 mM, applied to a 5-ml StrepTrap HP column, washed with binding buffer and eluted with 2.5 mM desthiobiotin. 5-fold excess copper was added as CuSO_4_, then unbound copper and desthiobiotin were removed by passing the protein in a HiLoad TM 16/600 Superdex 75 gel filtration column (Ge Healthcare) equilibrated with 10 mM sodium phosphate buffer pH 7.0. The protein was then concentrated using Microsep TM Advance Centrifugal Devices (Pall Corporation).

#### Substrates

Substrates used for the DNS assay, or PACE: barley β-glucan (β-D-1,3-1,4-glucan), mannan (borohydride reduced), konjac glucomannan (β-D-1,4), larch arabinogalactan, wheat arabinoxylan, tamarind seed xyloglucan, potato galactan and galactan (Gal:Ara:Rha:Xyl:GalUA = 91:2:1.7:0.3:5), are all purchased from Megazyme; locust bean gum (LBG), carboxymethyl-cellulose (CMC), microcrystalline cellulose (Avicel) and beech wood xylan are purchased from Sigma-Aldrich. Phosphoric acid swollen cellulose (PASC) was prepared as in [21]. Grass xylan (miscanthus stem alcohol-insoluble residues) was prepared as described in [74].

#### DNS-reducing sugar assays

The activity of *Lp*sGH5_8, *Lp*sGH11, *Lp*sGH134a and *Lp*sGH134b was determined by dinitrosalicylic acid (DNS)-reducing sugar assay on a range of polysaccharides (see paragraph “Substrates”). The 50-μl reactions were carried out in triplicates in 50 mM sodium phosphate buffer pH 6.0, 0.1% substrate and 3 μg of protein (0.5 μg for *Lp*sGH11). They were incubated at 30 °C for 2 h with shaking at 320 rpm and then 9 μl of the reaction was added to 31 μl of DNS reagent and heated at 100 °C for 5 min. After cooling at room temperature and addition of 160 μl water, the 540 nm absorbance was measured in a micro-plate reader and the results were compared to a glucose standard curve. The A_540_ of the substrates was subtracted from that of the samples. The DNS reagent was prepared by mixing 0.75 g of dinitrosalycilic acid, 1.4 g NaOH, 21.6 g sodium potassium tartrate tetrahydrate, 0.53 mL phenol and 0.59 g sodium metabisulfite in 100 ml of distilled water, and it was filtered and kept in the dark before used.

#### Product analysis by mass spectrometry (MS)

Reactions with the purified *Lp*sAA10A were carried out by mixing 4 mg mL − 1 substrate with purified copper-loaded enzyme (2 μM) and 4 mM electron donor (gallic acid), in 50 mM ammonium acetate buffer pH 6 in 2-mL plastic reaction tubes (reaction volume: 100 μL). The tubes were incubated for 24 h at 28 °C shaking at 1000 rpm, centrifuged at 14,000 rpm and the supernatant was collected for analysis through mass spectrometry. Briefly, 1 μl of supernatant was mixed with an equal volume of matrix solution (20 mg mL^− 1^ 2,5-dihydroxybenzoic acid (DHB) in 50% acetonitrile plus 0.1% TFA), spotted on a SCOUT-MTP 384 target plate (Bruker) and analysed by positive-mode MALDI-TOF MS using an Ultraflex III matrix-assisted laser desorption ionisation-time of flight/time of flight (MALDI/TOF-TOF) instrument (Bruker).

#### Polysaccharide analysis by carbohydrate gel electrophoresis (PACE)

##### Mannanase analysis

Purified enzyme at 20 μg/ml was mixed with 0.5% galactan, glucomannan, galactomannan, mannan or locust bean gum (LBG) or with 40 mg/ml of milled Scots pine wood (pre-treated in 0.5 N NaOH for 30 min at 90 °C and rinsed 5 times in 50 mM NaPO_4_ buffer) in 50 mM NaPO_4_ buffer pH 6.5 and incubated overnight at 30 °C with shaking. The samples were then centrifuged, supernatant was transferred to a new tube and undigested polysaccharides were removed by precipitation with 80% ethanol. Following centrifugation, supernatants were transferred to a new tube and dried.

##### Xylanase analysis

Miscanthus stem AIR (alcohol-insoluble residues) was pre-treated in 4 M NaOH for 1 h at RT and neutralised with HCl. Resultant substrate at 1 mg/ml (of initial untreated AIR) was digested overnight at RT with various amounts of xylanase (3–40 μg/ml). All samples were purified on Nanosep 10 K and dried. Dried digestion products and manno-oligosaccharide and xylo-oligosaccharide standards and appropriate controls were labelled with 8-aminonaphthalene-1,3,6-trisulfonic acid (ANTS; Invitrogen, www.invitrogen.com) and separated by polyacrylamide gels, as described previously [75]. PACE gels were visualised using a G-box (Syngene, www.syngene.com/). Experiments were carried out in triplicate, and the representative gels are shown.

## Supplementary Information

**Additional file 1: Fig. S1.** Scanning electron microscopic images of the gills and food groove of *L. pedicellatus*. A) Close up of the gills lamellae to show the numerous cilia that capture food and draw it to the food groove. B) Close up of the food groove to show the numerous cilia (most of the mucus is lost during critical point drying). File format .DOCX.

**Additional file 2: Fig. S2.** Searching for the opening of the duct of Deshayes in the shipworm digestive system. Left, transverse sections through the shell valve, from the anterior to posterior adductor muscle (A-E). Right, 3D rendered model of the whole shipworm, with the green highlighted region showing the position of the transverse cross section displayed on the left. File format .DOCX.

**Additional file 3: Fig. S3.** Scanning electron microscopy of *L. pedicellatus*. A dissected specimen with the mantle removed*,* showing the food groove connecting the gills to the mouth. File format .DOCX.

**Additional file 4: Fig. S4.** Bacterial symbionts in *L. pedicellatus*. A) TEM image of a gill bacteriocyte. Arrows indicate the numerous rod-shaped bacteria in the cell. B) Close-up view of gill bacteria (TEM). C) Detailed TEM showing a cross section of a gill bacterium and some of its features (periplasmic space and membranes). File format .DOCX.

**Additional file 5: Fig. S5.** SDS-PAGE and nitrocellulose western blot obtained by loading 0.6 μg of the recombinant purified bacterial *Lp*sGH5_8 (without the appended CBM). A) Coomassie Brilliant Blue stained SDS-PAGE gel of the purified protein. B) Western blot detection performed with the purified pre-immune serum. C) Western blot detection performed with the purified anti-protein serum. File format .DOCX.

**Additional file 6: Fig. S6.** Immunogold labelling of *Lps*GH5_8 in *L. pedicellatus* using pre-immune serum (negative control). A) SEM image illustrating the anatomical position of the gills, food groove, caecum and mouth in the shipworm body. B-D) Immunogold labelling of the lumen of the food groove (B), lumen of the caecum (C), and the gills (D) performed with pre-immune serum. No gold particles are observed in any of the samples. B = bacteria. File format .DOCX.

**Additional file 7: Table S1.** Details of the proteins identified through proteomic analysis of the crystalline style. The annotation was performed against the NCBI non-redundant database, the signal peptides were identified with the Signal P4.1 server and the CAZy domains were searched using dbCAN. Many of the contigs were out of frame or not full length (particularly the bacterial ones) and therefore the identification of the signal peptide was not possible. File format .DOCX.

**Additional file 8: Table S2.** CAZy families. List of the CAZy families mentioned in the results, with the description of the activities that have been recorded for enzymes listed in each family. The information has been gathered from the CAZy database (http://www.cazy.org). File format .DOCX.

**Additional file 9: Table S3.** CAZy families of the caecum. Table showing the relative abundance (calculated from the emPAI score) of eukaryotic and prokaryotic CAZy families identified in the proteomic analysis of the caecum content of *L. pedicellatus*. File format .DOCX.

**Additional file 10: Table S4.** Details of the proteins for which heterologous recombinant expression from symbionts cDNA was successful. The annotation was performed against the NCBI non-redundant database, the signal peptides were identified with the Signal P4.1 server and the CAZy domains were searched using dbCAN. Dis. bonds = disulfide bonds. File format .DOCX.

**Additional file 11: Table S5.** The amino acid sequences of the bacterial proteins *Lp*sGH5_8, *Lp*sGH11, *Lp*sGH134a, *Lp*sGH134b and *Lp*sAA10A. Text in red represents the signal peptide. File format .DOCX.

**Additional file 12: Fig. S7.** Characterisation of the recombinant bacterial CAZymes encoded by the endosymbionts. DNS reducing sugars assays showing activities on a number of substrates for *Lp*sGH5_8, *Lp*sGH11, *Lp*sGH134a and *Lp*sGH134b. CMC = *carboxymethyl cellulose*, LBG = locust bean gum. The nanomoles of sugars released by the different proteins cannot be compared quantitatively as different amounts were used for the assay. File format .DOCX.

**Additional file 13: Fig. S8.** MALDI-TOF MS analysis of *in vitro* activity assays with purified *Lp*sAA10A under the same experimental conditions as in Fig. 5 C. Panels from a to d show spectra of products obtained after incubation of the enzyme with 4 mg mL^-1^ Avicel (a), 4 mg mL^-1^ Avicel plus 4 mM gallic acid (b), 4 mg mL^-1^ Avicel plus 2 μM LPMO (c) and 4 mg mL^-1^ Avicel plus 2 μM LPMO and 4 mM gallic acid (d). In panels a to d, 100% relative intensity represents 1.3 × 10^4^ arbitrary units (a.u.). The panels from e to g show spectra of products obtained after incubation of 4 mg mL^-1^ PASC (e), 4 mg mL^-1^ PASC plus 4 mM gallic acid (f) and 4 mg mL^-1^ PASC plus 2 μM LPMO (g). In panels e to g, 100% relative intensity represents 1.0 × 10^4^ arbitrary units. File format .DOCX.

**Additional file 14: Table S6.** Primers used for the cloning of the bacterial proteins *Lp*sGH5_8, *Lp*sGH11, *Lp*sGH134a and *Lp*sGH134b. *Lp*sAA10A could not be amplified from the cDNA and therefore a synthetic version of the gene was codon-optimised for *E-coli* expression. File format .DOCX.

## Data Availability

All data generated or analysed during this study are included in this published article, its supplementary information files and publicly available repositories or are otherwise available in the following repositories: *Proteomics data*: Pesante, G., Sabbadin, F., Dowle, A-A. Analysis of the proteome of the crystalline style of the shipworm Lyrodus pedicellatus. MassIVE (Mass Spectrometry Interactive Virtual Environment). http://proteomecentral.proteomexchange.org/cgi/GetDataset?ID=PXD009432, http://massive.ucsd.edu/ProteoSAFe/dataset.jsp?task=57463a9d2e83481083eb47296f56a755, ftp://massive.ucsd.edu/MSV000082247/ *Transcriptomics data*: Pesante, G., Sabbadin, F., Li, Y. Lyrodus pedicellatus crystalline style sac RNA sequencing. NCBI (National Center for Biotechnology Information). BioProject ID PRJNA450378, SRA accession: SRP140494, www.ncbi.nlm.nih.gov/bioproject/450378. *Bacterial protein accessions:* Pesante, G., Sabbadin, F., Elias, L. Project PRJEB28739. ENA (European Nucleotide Archive), www.ebi.ac.uk/ena/data/view/LS999939-LS999943. *MicroCT data:* Shipway, J-R, Cragg, S-M. Proposed mechanism of enzyme transport between shipworms and their bacterial symbionts. Figshare. https://figshare.com/authors/Reuben_Shipway/5629772, DOI: 10.6084/m9.figshare.16635931.v1
