# Supplementary material for: Characterisation of the enzyme transport path between shipworms and their bacterial symbionts
Source: BMC Biol. 2021 Nov 1;19:233. doi: 10.1186/s12915-021-01162-6 (PMC8561940; doi:10.1186/s12915-021-01162-6)
Supplement: Supplementary file 1 — Additional file 1: Fig. S1. Scanning electron microscopic images of the gills and food groove of L. pedicellatus. A) Close up of the gills lamellae to show the numerous cilia that capture food and draw it to the food groove. B) Close up of the food groove to show the numerous cilia (most of the mucus is lost during critical point drying). File format .DOCX. [file 12915_2021_1162_MOESM1_ESM.docx]

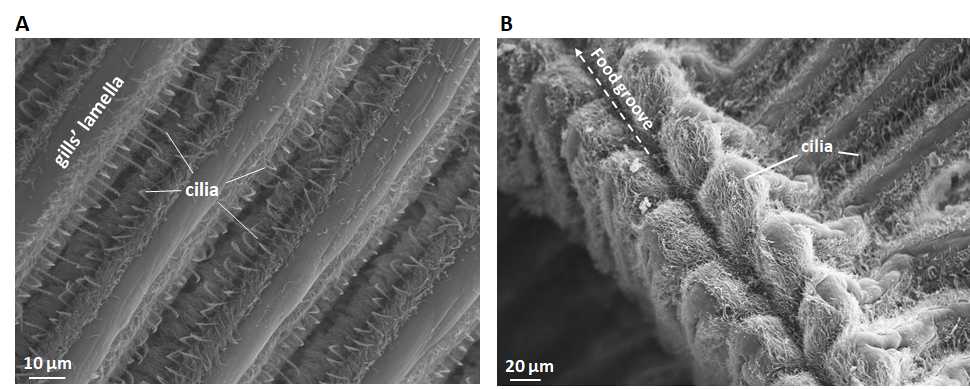


**Additional file 1. Scanning electron microscopic images of the gills and food groove of *L. pedicellatus*.** **A)** Close up of the gills lamellae to show the numerous cilia that capture food and draw it to the food groove. **B)** Close up of the food groove to show the numerous cilia (most of the mucus is lost during critical point drying).
