# Supplementary material for: Characterisation of the enzyme transport path between shipworms and their bacterial symbionts
Source: BMC Biol. 2021 Nov 1;19:233. doi: 10.1186/s12915-021-01162-6 (PMC8561940; doi:10.1186/s12915-021-01162-6)
Supplement: Supplementary file 2 — Additional file 2: Fig. S2. Searching for the opening of the duct of Deshayes in the shipworm digestive system. Left, transverse sections through the shell valve, from the anterior to posterior adductor muscle (A-E). Right, 3D rendered model of the whole shipworm, with the green highlighted region showing the position of the transverse cross section displayed on the left. File format .DOCX. [file 12915_2021_1162_MOESM2_ESM.docx]

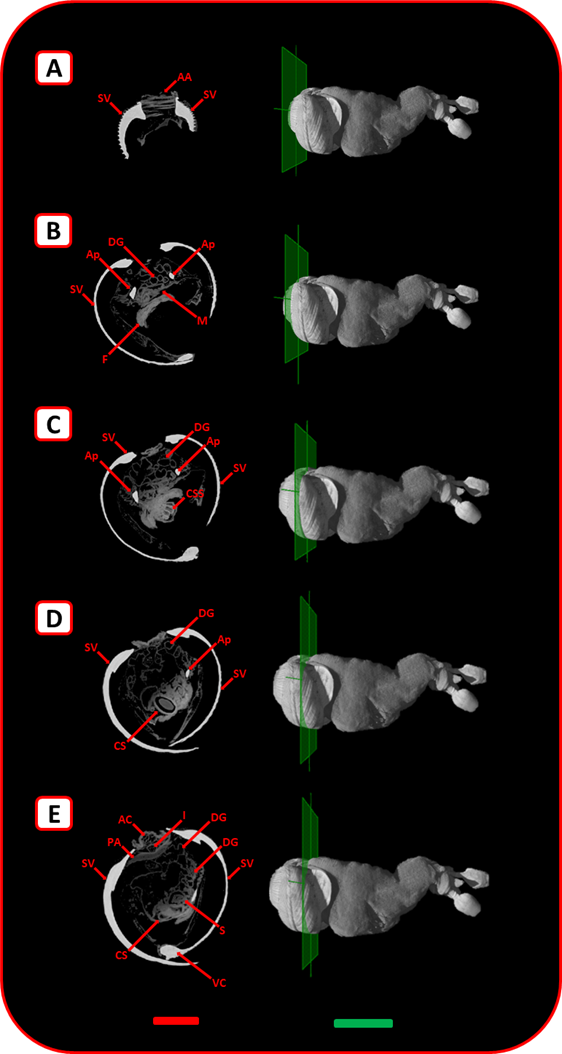


**Additional file 2. Searching for the opening of the duct of Deshayes in the shipworm digestive system.** Left, transverse sections through the shell valve, from the anterior to posterior adductor muscle (A-E). Right, 3D rendered model of the whole shipworm, with the green highlighted region showing the position of the transverse cross section displayed on the left. AA, anterior adductor muscle; AC, anal canal; Ap, apophysis; CS, crystalline style; CSS, crystalline style sac; DG, digestive glands; F, foot; I, intestine; M, mouth; PA, posterior adductor muscle; S, stomach; SV, shell valve; VC, ventral condoyle. Green and red scale bars = 5 mm and 1 cm, respectively.
