# Supplementary material for: Characterisation of the enzyme transport path between shipworms and their bacterial symbionts
Source: BMC Biol. 2021 Nov 1;19:233. doi: 10.1186/s12915-021-01162-6 (PMC8561940; doi:10.1186/s12915-021-01162-6)
Supplement: Supplementary file 3 — Additional file 3: Fig. S3. Scanning electron microscopy of L. pedicellatus. A dissected specimen with the mantle removed, showing the food groove connecting the gills to the mouth. File format .DOCX. [file 12915_2021_1162_MOESM3_ESM.docx]

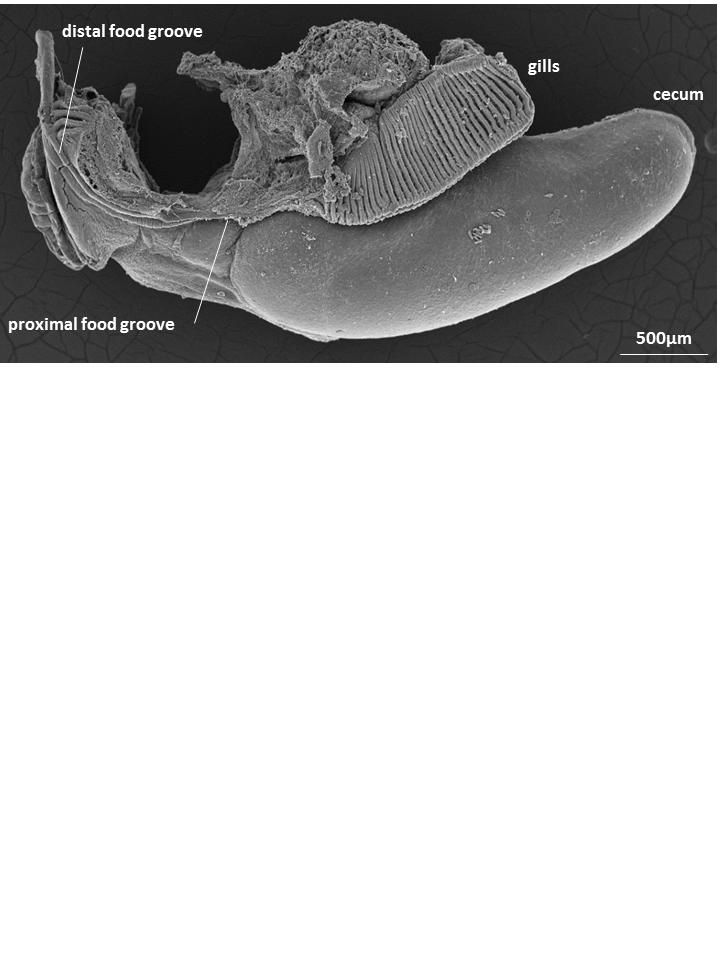


**Additional file 3**. **Scanning electron microscopy of *L. pedicellatus***. A dissected specimen with the mantle removed*,* showing the food groove connecting the gills to the mouth.
