# Supplementary material for: Characterisation of the enzyme transport path between shipworms and their bacterial symbionts
Source: BMC Biol. 2021 Nov 1;19:233. doi: 10.1186/s12915-021-01162-6 (PMC8561940; doi:10.1186/s12915-021-01162-6)
Supplement: Supplementary file 4 — Additional file 4: Fig. S4. Bacterial symbionts in L. pedicellatus. A) TEM image of a gill bacteriocyte. Arrows indicate the numerous rod-shaped bacteria in the cell. B) Close-up view of gill bacteria (TEM). C) Detailed TEM showing a cross section of a gill bacterium and some of its features (periplasmic space and membranes). File format .DOCX. [file 12915_2021_1162_MOESM4_ESM.docx]

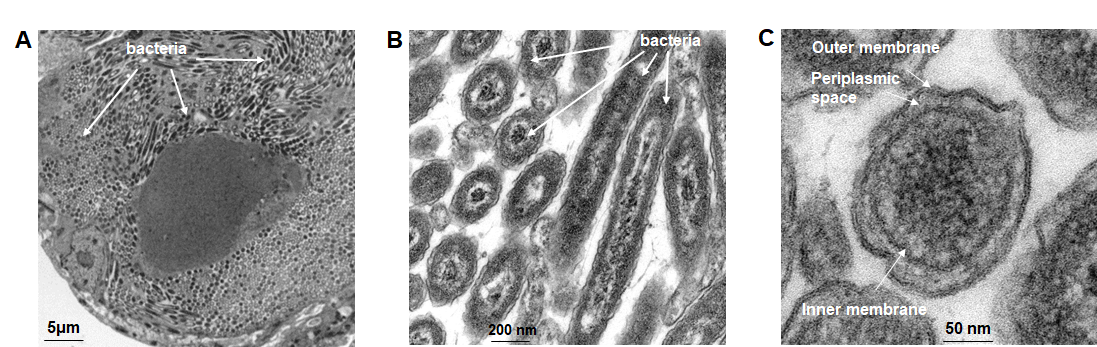


**Additional file 4. Bacterial symbionts in *L. pedicellatus*.** **A)** TEM image of a gill bacteriocyte. Arrows indicate the numerous rod-shaped bacteria in the cell. **B)** Close-up view of gill bacteria (TEM). **C)** Detailed TEM showing a cross section of a gill bacterium and some of its features (periplasmic space and membranes).
