# Supplementary material for: Characterisation of the enzyme transport path between shipworms and their bacterial symbionts
Source: BMC Biol. 2021 Nov 1;19:233. doi: 10.1186/s12915-021-01162-6 (PMC8561940; doi:10.1186/s12915-021-01162-6)
Supplement: Supplementary file 5 — Additional file 5: Fig. S5. SDS-PAGE and nitrocellulose western blot obtained by loading 0.6 μg of the recombinant purified bacterial LpsGH5_8 (without the appended CBM). A) Coomassie Brilliant Blue stained SDS-PAGE gel of the purified protein. B) Western blot detection performed with the purified pre-immune serum. C) Western blot detection performed with the purified anti-protein serum. File format .DOCX. [file 12915_2021_1162_MOESM5_ESM.docx]

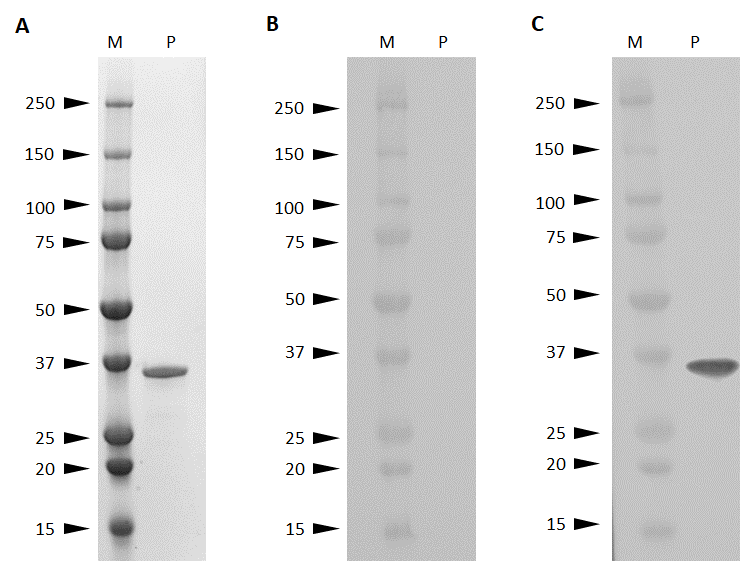


**Additional file 5. SDS-PAGE and nitrocellulose western blot obtained by loading 0.6 µg of the recombinant purified bacterial *Lp*sGH5_8 (without the appended CBM). A)** Coomassie Brilliant Blue stained SDS-PAGE gel of the purified protein. **B)** Western blot detection performed with the purified pre-immune serum. **C)** Western blot detection performed with the purified anti-protein serum. Both sera were used at a dilution of 1:100, while the secondary antibody (stabilised goat anti-rabbit IgG (H+L) peroxidase-conjugated antibodies from Pierce) was used with a dilution factor of 1:500. Protein visualisation for the western blot was performed with the SuperSignal^TM^ West Pico PLUS chemiluminescent substrate (Thermo Scientific) using the Syngene PXi gel documentation imaging system (the latter also used for to visualise the Coomassie stained gel)**.** Both SDS-PAGE and western blot with the anti-protein serum show a clean band just under the 37 kDa mark, which corresponds to the predicted molecular weight (34 kDa) for the mature *Lp*sGH5_8. No signal is seen for the pre-immune serum. M: protein marker. P: purified protein.
