# Supplementary material for: Characterisation of the enzyme transport path between shipworms and their bacterial symbionts
Source: BMC Biol. 2021 Nov 1;19:233. doi: 10.1186/s12915-021-01162-6 (PMC8561940; doi:10.1186/s12915-021-01162-6)
Supplement: Supplementary file 6 — Additional file 6: Fig. S6. Immunogold labelling of LpsGH5_8 in L. pedicellatus using pre-immune serum (negative control). A) SEM image illustrating the anatomical position of the gills, food groove, caecum and mouth in the shipworm body. B-D) Immunogold labelling of the lumen of the food groove (B), lumen of the caecum (C), and the gills (D) performed with pre-immune serum. No gold particles are observed in any of the samples. B = bacteria. File format .DOCX. [file 12915_2021_1162_MOESM6_ESM.docx]

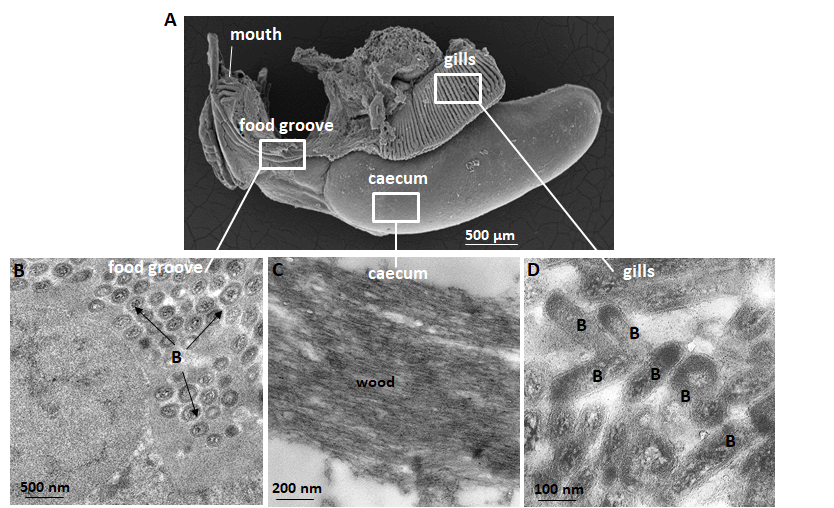


**Additional file 6. Immunogold labelling of *Lps*GH5_8 in *L. pedicellatus* using pre-immune serum (negative control). A)** SEM image illustrating the anatomical position of the gills, food groove, caecum and mouth in the shipworm body. **B-D)** Immunogold labelling of the lumen of the food groove **(B)**, lumen of the caecum **(C),** and the gills **(D)** performed with pre-immune serum. No gold particles are observed in any of the samples. B = bacteria.
