# Supplementary material for: Characterisation of the enzyme transport path between shipworms and their bacterial symbionts
Source: BMC Biol. 2021 Nov 1;19:233. doi: 10.1186/s12915-021-01162-6 (PMC8561940; doi:10.1186/s12915-021-01162-6)
Supplement: Supplementary file 7 — Additional file 7: Table S1. Details of the proteins identified through proteomic analysis of the crystalline style. The annotation was performed against the NCBI non-redundant database, the signal peptides were identified with the Signal P4.1 server and the CAZy domains were searched using dbCAN. Many of the contigs were out of frame or not full length (particularly the bacterial ones) and therefore the identification of the signal peptide was not possible. File format .DOCX. [file 12915_2021_1162_MOESM7_ESM.docx]

**Additional file 7. Details of the proteins identified through proteomic analysis of the crystalline style**. The annotation was performed against the NCBI non-redundant database, the signal peptides were identified with the Signal P4.1 server and the CAZy domains were searched using dbCAN. Many of the contigs were out of frame or not full length (particularly the bacterial ones) and therefore the identification of the signal peptide was not possible.

| **Contig name** | **Eukaryotic/**  **prokaryotic** | **Signal**  **peptide** | **Annotation** | **Species** | **E-value** | **CAZy domains** |
| --- | --- | --- | --- | --- | --- | --- |
| c165315_g1_i2 | prokaryotic | yes | GH family 5_8 domain-containing protein | *Alteromonadaceae bacterium Bs08* | 2.00E-46 | GH5_8+CBM10+CBM2 |
| c122374_g1_i1 | prokaryotic | yes | plant cell wall polysaccharide active protein | *Alteromonadaceae bacterium Bs08* | 1.00E-56 | GH134+3CBM10 |
| c151620_g1_i1 | eukaryotic | no | endoglucanase 13-like | *Mizuhopecten yessoensis* | 3.00E-51 | GH9 |
| c293208_g1_i1 | prokaryotic | no | beta-mannosidase | *Teredinibacter sp. 1162T.S.0a.05* | 4.00E-28 | GH5 |
| c169024_g2_i1 | eukaryotic | no | mannan endo-1,4-beta-mannosidase | *Mizuhopecten yessoensis* | 3.00E-128 | GH5_10 |
| c154126_g1_i1 | prokaryotic | no | glycosyl hydrolase family protein | *Zobellia galactanivorans* | 1.00E-44 | GH16 |
| c180176_g1_i1 | prokaryotic | yes | plant cell wall polysaccharide active protein | *Alteromonadaceae bacterium Bs12* | 1.00E-104 | GH134+3CBM10 |
| c179074_g2_i2 | eukaryotic | no | chitobiase | *Mizuhopecten yessoensis* | 3.00E-51 | GH20 |
| c176756_g1_i11 | eukaryotic | yes | endo-1,3-beta-D-glucanase | *Tapes literata* | 1.00E-146 | GH16 |
| c179423_g1_i1 | prokaryotic | yes | carbohydrate esterase family 3 | *Teredinibacter turnerae T7901* | 2.00E-92 | CE3+CBM10 |
| c170561_g7_i1 | prokaryotic | no | GH family 10 domain-containing protein | *Alteromonadaceae bacterium Bs02* | 5.00E-73 | GH10 |
| c177884_g1_i11 | eukaryotic | no | beta-galactosidase-1-like protein 2 | *Mizuhopecten yessoensis* | 2.00E-131 | GH35 |
| c177001_g3_i1 | prokaryotic | no | beta-mannosidase | *Teredinibacter sp. 1162T.S.0a.05* | 2.00E-109 | GH5+CBM10+CBM2 |
| c178040_g2_i1 | eukaryotic | yes | multidomain GH1 | *Lyrodus pedicellatus* | 0 | GH1 |
| c178224_g1_i7 | eukaryotic | yes | multidomain GH1 | *Lyrodus pedicellatus* | 9.00E-108 | GH1 |
| c170561_g5_i1 | prokaryotic | no | acetyl xylan esterase | *Alteromonadales bacterium BS08* | 8.00E-146 | GH10 |
| c86197_g1_i1 | eukaryotic | yes | endoglucanase-like | *Crassostrea gigas* | 6.00E-43 | GH45 |
| c173097_g2_i4 | eukaryotic | yes | Endoglucanase E-4 | *Crassostrea gigas* | 0.00 | GH9+CBM2 |
| c175047_g4_i1 | eukaryotic | yes | multidomain GH1 | *Lyrodus pedicellatus* | 0 | GH1 |
| c176917_g2_i1 | prokaryotic | no | GH family 5_53 domain-containing protein | *Alteromonadaceae bacterium Bs02* | 2.00E-179 | GH5_53+CBM10+CBM2 |
| c177442_g1_i2 | prokaryotic | no | glycoside hydrolase family 9 | *Alteromonadales bacterium BS08* | 0.00 | GH9+CBM3+CBM10 |
| c180241_g3_i2 | prokaryotic | no | GH family 5_53 domain-containing protein | *Alteromonadaceae bacterium Bs12* | 0 | GH5_53+CBM10 |
| c178040_g3_i1 | eukaryotic | yes | multidomain GH1 | *Lyrodus pedicellatus* | 0 | GH1 |
| c178040_g1_i1 | eukaryotic | yes | multidomain GH1 | *Lyrodus pedicellatus* | 0 | GH1+GH1 |
| c178089_g2_i3 | eukaryotic | no | beta-mannosidase-like | *Mizuhopecten yessoensis* | 0.00 | GH2 |
