# Supplementary material for: Characterisation of the enzyme transport path between shipworms and their bacterial symbionts
Source: BMC Biol. 2021 Nov 1;19:233. doi: 10.1186/s12915-021-01162-6 (PMC8561940; doi:10.1186/s12915-021-01162-6)
Supplement: Supplementary file 8 — Additional file 8: Table S2. CAZy families. List of the CAZy families mentioned in the results, with the description of the activities that have been recorded for enzymes listed in each family. The information has been gathered from the CAZy database (http://www.cazy.org). File format .DOCX. [file 12915_2021_1162_MOESM8_ESM.docx]

**Additional file 8.** **CAZy families**. List of the CAZy families mentioned in the results, with the description of the activities that have been recorded for enzymes listed in each family. The information has been gathered from the CAZy database (http://www.cazy.org).

| **CAZy family** | **Recorded activities** |
| --- | --- |
| AA10 | chitin or cellulose copper-dependent lytic polysaccharide monooxygenase |
| CE3 | acetyl xylan esterase |
| GH1 | β-glucosidase, β-galactosidase, β-mannosidase, β-glucuronidase, β-xylosidase, β-D-fucosidase, phlorizin hydrolase, exo-β-1,4-glucanase, 6-phospho-β-galactosidase, 6-phospho-β-glucosidase, strictosidine β-glucosidase, lactase, amygdalin β-glucosidase, prunasin β-glucosidase, vicianin hydrolase, raucaffricine β-glucosidase, thioglucosidase, β-primeverosidase, isoflavonoid 7-O-β-apiosyl-β-glucosidase, ABA-specific β-glucosidase, DIMBOA β-glucosidase, β-glycosidase, hydroxyisourate hydrolase |
| GH2 | β-galactosidase, β-mannosidase, β-glucuronidase, α-L-arabinofuranosidase, mannosylglycoprotein endo-β-mannosidase, exo-β-glucosaminidase, α-L-arabinopyranosidase, β-galacturonidase |
| GH5 | endo-β-1,4-glucanase/cellulase, endo-β-1,4-xylanase, β-glucosidase, β-mannosidase, β-glucosylceramidase, glucan β-1,3-glucosidase, licheninase, exo-β-1,4-glucanase/cellodextrinase, glucan endo-1,6-β-glucosidase, mannan endo-β-1,4-mannosidase, cellulose β-1,4-cellobiosidase, steryl β-glucosidase, endoglycoceramidase, chitosanase, β-primeverosidase, xyloglucan-specific endo-β-1,4-glucanase, endo-β-1,6-galactanase, hesperidin 6-O-α-L-rhamnosyl-β-glucosidase, β-1,3-mannanase, arabinoxylan-specific endo-β-1,4-xylanase, mannan transglycosylase |
| GH7 | endo-β-1,4-glucanase, reducing end-acting cellobiohydrolase, chitosanase, endo-β-1,3-1,4-glucanase |
| GH9 | endoglucanase, endo-β-1,3(4)-glucanase/lichenase-laminarinase, β-glucosidase, lichenase/endo-β-1,3-1,4-glucanase, exo-β-1,4-glucanase/cellodextrinase, cellobiohydrolase, xyloglucan-specific endo-β-1,4-glucanase/endo-xyloglucanase, exo-β-glucosaminidase |
| GH10 | endo-1,4-β-xylanase, endo-1,3-β-xylanase, tomatinase, xylan endotransglycosylase |
| GH11 | endo-β-1,4-xylanase, endo-β-1,3-xylanase |
| GH16 | xyloglucan/xyloglucosyltransferase, keratan-sulfate endo-1,4-β-galactosidase, endo-1,3-β-glucanase, endo-1,3(4)-β-glucanase, licheninase, β-agarase, κ-carrageenase, xyloglucanase, endo-β-1,3-galactanase, β-porphyranase, hyaluronidase, endo-β-1,4-galactosidase, chitin β-1,6-glucanosyltransferase, endo-β-1,4-galactosidase |
| GH20 | β-hexosaminidase, lacto-N-biosidase, β-1,6-N-acetylglucosaminidase), β-6-SO3-N-acetylglucosaminidase |
| GH35 | β-galactosidase, exo-β-glucosaminidase, exo-β-1,4-galactanase, β-1,3-galactosidase |
| GH45 | endoglucanase |
| GH134 | endo-β-1,4-mannanase |
| **Associated modules** |  |
| CBM2 | cellulose, chitin or xylan-binding |
| CBM10 | cellulose-binding |
