# Supplementary material for: Characterisation of the enzyme transport path between shipworms and their bacterial symbionts
Source: BMC Biol. 2021 Nov 1;19:233. doi: 10.1186/s12915-021-01162-6 (PMC8561940; doi:10.1186/s12915-021-01162-6)
Supplement: Supplementary file 9 — Additional file 9: Table S3. CAZy families of the caecum. Table showing the relative abundance (calculated from the emPAI score) of eukaryotic and prokaryotic CAZy families identified in the proteomic analysis of the caecum content of L. pedicellatus. File format .DOCX. [file 12915_2021_1162_MOESM9_ESM.docx]

**Additional file 9. CAZy families of the caecum.** Table showing the relative abundance (calculated from the emPAI score) of eukaryotic and prokaryotic CAZy families identified in the proteomic analysis of the caecum content of *L. pedicellatus*.
