# Supplementary material for: Characterisation of the enzyme transport path between shipworms and their bacterial symbionts
Source: BMC Biol. 2021 Nov 1;19:233. doi: 10.1186/s12915-021-01162-6 (PMC8561940; doi:10.1186/s12915-021-01162-6)
Supplement: Supplementary file 10 — Additional file 10: Table S4. Details of the proteins for which heterologous recombinant expression from symbionts cDNA was successful. The annotation was performed against the NCBI non-redundant database, the signal peptides were identified with the Signal P4.1 server and the CAZy domains were searched using dbCAN. Dis. bonds = disulfide bonds. File format .DOCX. [file 12915_2021_1162_MOESM10_ESM.docx]

**Additional file 10. Details of the proteins for which heterologous recombinant expression from symbionts cDNA was successful.** The annotation was performed against the NCBI non-redundant database, the signal peptides were identified with the Signal P4.1 server and the CAZy domains were searched using dbCAN. Dis. bonds= disulfide bonds.

| **Contig name** | **Eukaryotic/**  **prokaryotic** | **Signal**  **peptide** | **Annotation** | **Species** | **E-value** | **CAZy domains** | **Dis.**  **bonds** | **Name in paper** |
| --- | --- | --- | --- | --- | --- | --- | --- | --- |
| c177001_g3_i1 | prokaryotic | yes | GH5_8 domain-containing protein | *Alteromonadaceae bacterium Bs08* | 3e-174 | GH5_8+CBM10 | 5 | *Lp*sGH5_8 |
| c169869_g1_i2 | prokaryotic | yes | 1,4-beta-xylanase | *Teredinibacter sp. 1162T.S.0a.05* | 2e-83 | GH11+CBM10+CBM5 | 4 | *Lp*sGH11 |
| c122374_g1_i1 | prokaryotic | yes | plant cell wall polysaccharide active protein | *Alteromonadaceae bacterium Bs12* | 2e-127 | GH134+3CBM10 | 10 | *Lp*sGH134b |
| c180176_g1_i1 | prokaryotic | yes | plant cell wall polysaccharide active protein | *Alteromonadaceae bacterium Bs12* | 7e-104 | GH134+3CBM10 | 10 | *Lp*sGH134a |
| c173837_g2_i2 | prokaryotic | yes | auxiliary activity family 10 domain-containing protein | *Alteromonadaceae bacterium Bs12* | 6e-92 | AA10+CBM10 | 8 | *Lp*sAA10 |
