# Supplementary material for: Characterisation of the enzyme transport path between shipworms and their bacterial symbionts
Source: BMC Biol. 2021 Nov 1;19:233. doi: 10.1186/s12915-021-01162-6 (PMC8561940; doi:10.1186/s12915-021-01162-6)
Supplement: Supplementary file 11 — Additional file 11: Table S5. The amino acid sequences of the bacterial proteins LpsGH5_8, LpsGH11, LpsGH134a, LpsGH134b and LpsAA10A. Text in red represents the signal peptide. File format .DOCX. [file 12915_2021_1162_MOESM11_ESM.docx]

**Additional file 11. The amino acid sequences of the bacterial proteins *Lp*sGH5_8, *Lp*sGH11, *Lp*sGH134a, *Lp*sGH134b and *Lp*sAA10A**. Text in red represents the signal peptide.

| ***Lp*sGH5_8** |
| --- |
| MSVVYSGLSIKNNNGNKIMLVNRLKHRLGAGIKNSLAAVAIPFLAAISFNANAGFSVSGSQLLDANGQSFVMRGVNHPHAWYTGETGSFADIAATGANTVRVVLSNGHQWTRNSASDVANVISLCKQNQLICVLEVHDATGGGEEGAAGTIAGAAQYWVDIASALQGEEDYVIINIANEPVGNNQPSSMWINDHISAIQKLRNAGLTHTLMIDAPNWGQDWENVMRNNAPQVAAADSLNNTMFSVHMYQVYQDYSTINGYLTGFVSDHNLPIIVGEFGGDHQGDEVDEDSILQVAQQNGIGYLGWSWSGNGDCCTTLDIVNNFNGSSLTSWGNRLINGTNGIAQTSQRASVYGGGSTTSSSSSSSSSSSNNSSSSSSSSSSSSSSSSSSSSGGGACTECNWYGSTYDLCNNQNSGWGWENQQSCIGIDTCEGQNGNGGPICSGS- |
| ***Lp*sGH11** |
| MKVTDFKSGYVGFAKTVSFALVATAAALFSIGANAQTTLSSNSTGNNDGYYYTFWKDSGDASMTLYPGGRYTSSWTSSTNNWVGGYGWNPGSRRTVSYSGSYSANGTSYLALYGWTRNPLIEYYVVENWVNYNPSSGADTYGTVNIDGSTYTLARSQRVQQPSIDGTATFYQYWSVRSNKRTSGTIDVGAHFDAWASVGLNLGTEFNYMVMATEGYQSGGSSDITVSQGGSSGSTTSSSSSSSSSSSSSSSSSGSTGGQCGNCNWYGSIYPLCNNQNSGWGWESNQSCIGVDTCSGQNGNGGPVDCNGGGSNSSSSSSSSSSSSSSSSNSSSSSSSSSSSSSSSSSSSSSSGGNNGGGTNCGGVNEYPNWTAKDWSGGTNNHANSGDQMVYQGQLYQANWYTSTVPGSDASWTTVGSCN- |
| ***Lp*sGH134a** |
| MKPIIKVVFMFVIYILSSQSFAQSQCNWYGTNYPVCQNQSTEWGWENNQSCIGPETCAANGGSSSGGSSSSSSSSSSSSSTGGSGGAAGGQCDWYGSNYPLCTNQNSGWGWENNQSCIGPDTCTNPSGSSGGGSGNSSSSSSSSSSSSSSSTGGSASGGTCNWYGTDHPMCVNTASGWGWENNQSCISQAECGTQGGSGGSSGGGNTGGGSNNGGSAGGSCGVGTCPDSLSCPGGASCGCYTVSGLGANKRAYQDAGADRRFLASAMMETELMDTNYTYGDGKSGDAFNAGATKQNWGMIRQCHSAWSGYGSGDYAVSDQMNSSRSLDVQVYGECRSYFGNNWFAGHRNGSSGLENPNTSDIQRFKEGYDWTYNMLNGHECDDVRFWVDIPAI- |
| ***Lp*sGH134b** |
| MGANLSCWLFRASTFLGGNTRYSHIHNRSFFMKPIIKVVFMFVIYLLSSQSFAQSQCDWYGTNYPVCQNQSSEWGWENNQSCIGPETCAANGGSSSGGSGGGSSSSSSSSSSSSSSSTGGSGGAAGGQCDWYGSNYPLCSNQNSGWGWENNQSCIGPDTCTSPSGSSGSGSGNSSSSSSSSSSSSSSSSTGGSANGGTCNWYGTNHPMCVNTNSGWGYENNQSCISQAECDTQGGSGSSSSGGNTGGGSNNGGGSTGGGSCGVGTCPDSLNCPGGASCGCYTVSGLGANKRAYQDAGADRRFLASAMMETELMDTNYTYGDGKSGDAFNAGATKQNWGMIRQCHSAWSGYGADDYSVSDEMNSSRSLDVQVYGECRSYFGNNWFAGHRNGSSGLDNPNTDDIQRFREGYDWTYNMLNGHECDDVRFWVDIPAI- |
| ***Lp*sAA10A** |
| MFATRTIKATAALALLGFAGMAFGHGYIESPPSRQQHCGVEVKPDNRSSNKCDEAFDNYGGQSSHWYNFMSVVAHNEGRKVEKGTTNVCGFDGESFNPAPWDTAADWPTTAISAGNQNFVWNISNGPHFSDTKELVFWITKPGFTFDSNRVLTWDDFEPAPFCDEQVGSGDFGSNPNVTAGSVDITVNCNVPSRSGRHVIYSEWGRDPATYERFFSCVDVTFGGSNGGGSTTSSSSSSSSSSSSSSSSSSSSNSSSSSSSSSSSSSSSSGSTGGQCVCQWYSEGTYPICENSTGWGWENNQSCQVASGGSSGGGSSSGSCGTGSCPSSLSCPSGMNCGCYTVFRFGSKQTRLSRCRGRSKLPRVSNDGNRSDGYQLRLW- |
