# Supplementary material for: Characterisation of the enzyme transport path between shipworms and their bacterial symbionts
Source: BMC Biol. 2021 Nov 1;19:233. doi: 10.1186/s12915-021-01162-6 (PMC8561940; doi:10.1186/s12915-021-01162-6)
Supplement: Supplementary file 12 — Additional file 12: Fig. S7. Characterisation of the recombinant bacterial CAZymes encoded by the endosymbionts. DNS reducing sugars assays showing activities on a number of substrates for LpsGH5_8, LpsGH11, LpsGH134a and LpsGH134b. CMC = carboxymethyl cellulose, LBG = locust bean gum. The nanomoles of sugars released by the different proteins cannot be compared quantitatively as different amounts were used for the assay. File format .DOCX. [file 12915_2021_1162_MOESM12_ESM.docx]

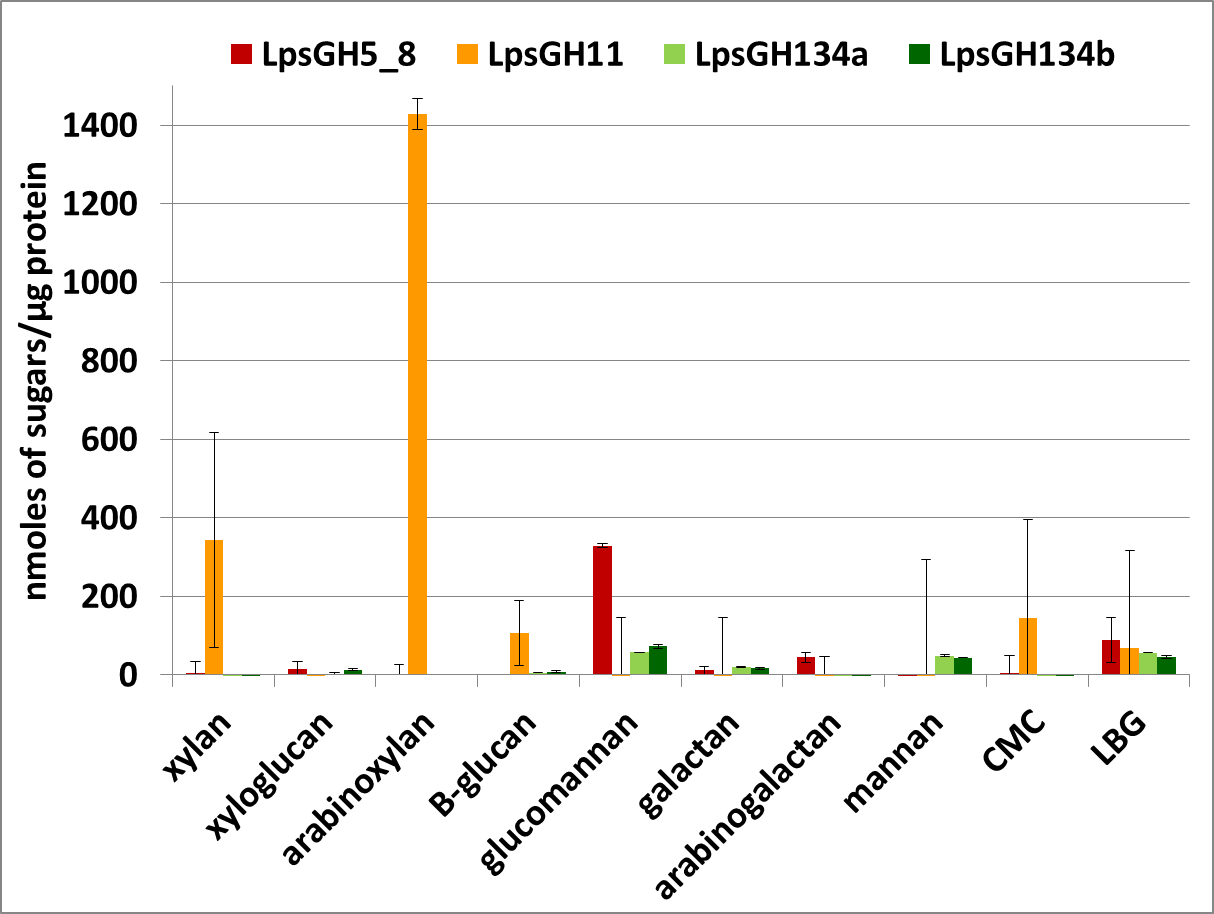


|  |  | *Lps*GH5_8 | | | | | | *Lps*GH11 | | | | |  |
| --- | --- | --- | --- | --- | --- | --- | --- | --- | --- | --- | --- | --- | --- |
|  | **Rep 1** | | **Rep 2** | **Rep 3** | **Mean** | **St. dev.** | **Rep 1** | | **Rep 2** | **Rep 3** | **Mean** | **St. dev.** | |
| Substrate | **nm/μg** | | **nm/μg** | **nm/μg** | **nm/μg** | **nm/μg** | **nm/μg** | | **nm/μg** | **nm/μg** | **nm/μg** | **nm/μg** | |
| Xylan | -30.68 | | 15.34 | 25.35 | 3.34 | 29.88 | 122.18 | | 649.09 | 259.64 | 343.64 | 273.31 | |
| Xyloglucan | -4.00 | | 12.01 | 34.02 | 14.01 | 19.09 | -916.08 | | -252.00 | -389.45 | -519.18 | 350.53 | |
| Arabinoxylan | 26.68 | | -3.34 | -23.35 | 0.00 | 25.18 | 1381.75 | | 1450.90 | 1450.90 | 1427.85 | 39.92 | |
| B-glucan | **-** | | **-** | **-** | **-** | **-** | 83.97 | | 38.18 | 198.54 | 106.90 | 82.60 | |
| Glucomannan | 327.52 | | 333.52 | 325.51 | 328.85 | 4.17 | -61.07 | | -267.27 | 145.09 | -61.08 | 206.18 | |
| Galactan | 14.67 | | -3.34 | 22.68 | 11.34 | 13.32 | -221.39 | | -15.27 | -381.82 | -206.16 | 183.75 | |
| Arabinogalactan | 33.35 | | 33.35 | 65.37 | 44.02 | 18.49 | -526.75 | | 22.91 | 91.64 | -137.40 | 338.93 | |
| Mannan | 8.67 | | -11.34 | -75.38 | -26.01 | 43.90 | 38.17 | | -328.36 | 152.73 | -45.82 | 251.30 | |
| CMC | -18.01 | | -38.02 | 70.04 | 4.67 | 57.49 | -137.41 | | 320.73 | 252.00 | 145.10 | 247.07 | |
| LBG | 96.72 | | 82.71 | 86.71 | 88.72 | 7.22 | 7.63 | | 213.82 | -15.27 | 68.73 | 126.17 | |
|  |  | |  |  |  |  |  | |  |  |  |  | |
|  | ***Lps*GH134a** | | | | | | ***Lps*GH134b** | | | | | | |
|  | **Rep 1** | | **Rep 2** | **Rep 3** | **Mean** | **St. dev.** | **Rep 1** | | **Rep 2** | **Rep 3** | **Mean** | **St. dev.** | |
| Substrate | **nm/μg** | | **nm/μg** | **nm/μg** | **nm/μg** | **nm/μg** | **nm/μg** | | **nm/μg** | **nm/μg** | **nm/μg** | **nm/μg** | |
| Xylan | -1.57 | | 0.26 | -0.26 | -0.52 | 0.94 | -1.57 | | 0.26 | -0.26 | -0.52 | 0.94 | |
| Xyloglucan | 6.29 | | 7.34 | 4.72 | 6.11 | 1.32 | 6.29 | | 7.34 | 4.72 | 6.11 | 1.32 | |
| Arabinoxylan | **-** | | **-** | **-** | **-** |  | **-** | | **-** | **-** | **-** |  | |
| B-glucan | 4.19 | | 2.10 | 4.45 | 3.58 | 2.75 | 4.19 | | 2.10 | 4.45 | 3.58 | 2.75 | |
| Glucomannan | 38.77 | | 36.15 | 33.27 | 36.07 | 1.32 | 38.77 | | 36.15 | 33.27 | 36.07 | 1.32 | |
| Galactan | 7.47 | | 7.73 | 8.51 | 7.90 | 0.55 | 7.47 | | 7.73 | 8.51 | 7.90 | 0.55 | |
| Arabinogalactan | -1.44 | | -1.70 | -1.96 | -1.70 | 0.26 | -1.44 | | -1.70 | -1.96 | -1.70 | 0.26 | |
| Mannan | 24.23 | | 20.30 | 20.83 | 21.79 | 2.13 | 24.23 | | 20.30 | 20.83 | 21.79 | 2.13 | |
| CMC | -2.75 | | -0.92 | -4.85 | -2.84 | 1.97 | -2.75 | | -0.92 | -4.85 | -2.84 | 1.97 | |
| LBG | 24.76 | | 26.07 | 18.21 | 23.01 | 4.21 | 24.76 | | 26.07 | 18.21 | 23.01 | 4.21 | |

**Additional file 12. Characterisation of the recombinant bacterial CAZymes encoded by the endosymbionts.** DNS reducing sugars assays showing activities on a number of substrates for *Lp*sGH5_8, *Lp*sGH11, *Lp*sGH134a and *Lp*sGH134b. CMC = carboxymethyl cellulose, LBG=locust bean gum. The nanomoles of sugars released by the different proteins cannot be compared quantitatively as different amounts were used for the assay.
