# Supplementary material for: Characterisation of the enzyme transport path between shipworms and their bacterial symbionts
Source: BMC Biol. 2021 Nov 1;19:233. doi: 10.1186/s12915-021-01162-6 (PMC8561940; doi:10.1186/s12915-021-01162-6)
Supplement: Supplementary file 13 — Additional file 13: Fig. S8. MALDI-TOF MS analysis of in vitro activity assays with purified LpsAA10A under the same experimental conditions as in Fig. 5 C. Panels from a to d show spectra of products obtained after incubation of the enzyme with 4 mg mL-1 Avicel (a), 4 mg mL-1 Avicel plus 4 mM gallic acid (b), 4 mg mL-1 Avicel plus 2 μM LPMO (c) and 4 mg mL-1 Avicel plus 2 μM LPMO and 4 mM gallic acid (d). In panels a to d, 100% relative intensity represents 1.3 × 10^4^ arbitrary units (a.u.). The panels from e to g show spectra of products obtained after incubation of 4 mg mL-1 PASC (e), 4 mg mL-1 PASC plus 4 mM gallic acid (f) and 4 mg mL-1 PASC plus 2 μM LPMO (g). In panels e to g, 100% relative intensity represents 1.0 × 10^4^ arbitrary units. File format .DOCX. [file 12915_2021_1162_MOESM13_ESM.docx]

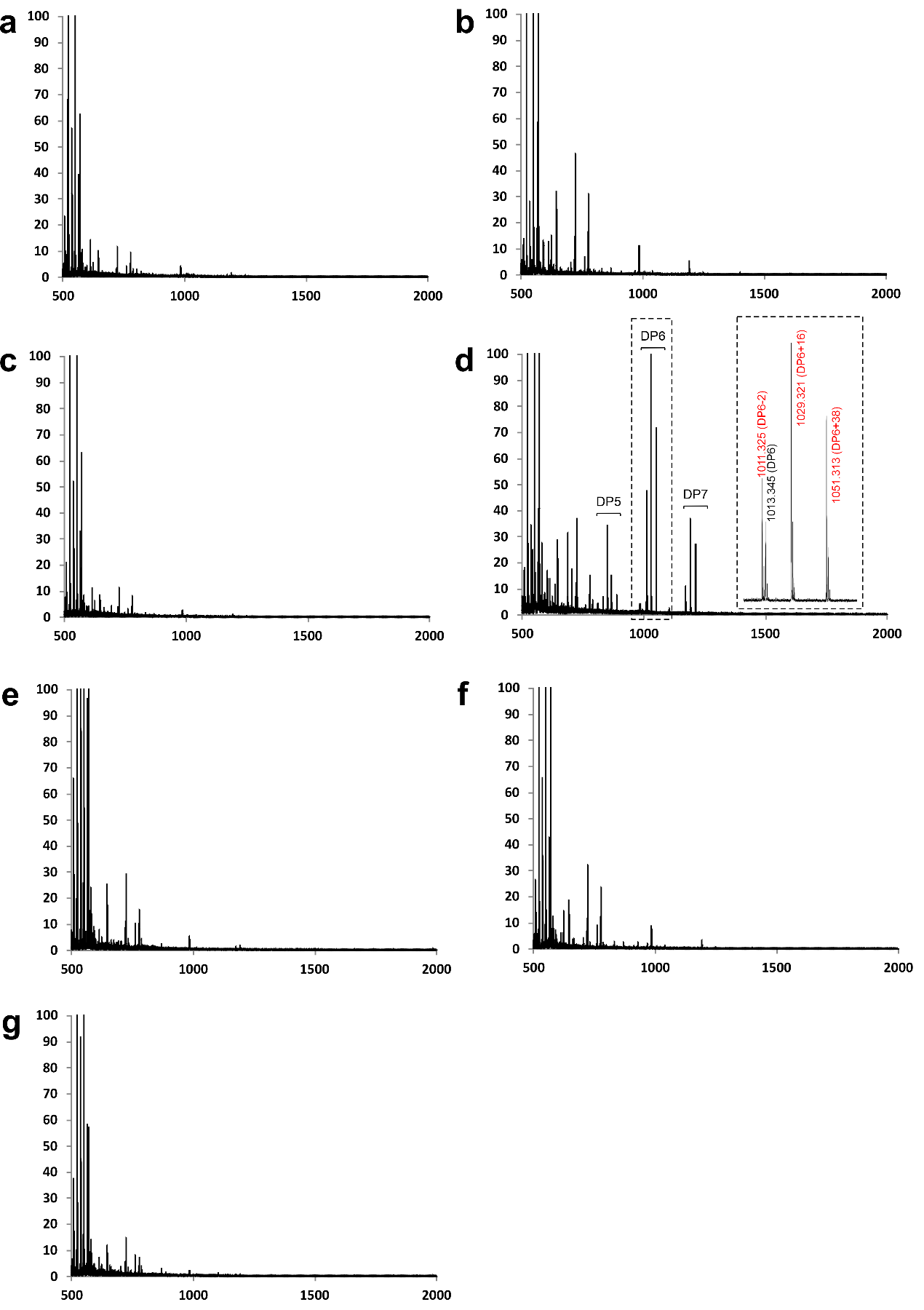


**Additional file 13. MALDI-TOF MS analysis of *in vitro* activity assays with purified *Lp*sAA10A under the same experimental conditions as in Fig. 4 C**. Panels from a to d show spectra of products obtained after incubation of the enzyme with 4 mg mL^-1^ Avicel (**a**), 4 mg mL^-1^ Avicel plus 4 mM gallic acid (**b**), 4 mg mL^-1^ Avicel plus 2 µM LPMO (**c**) and 4 mg mL^-1^ Avicel plus 2 µM LPMO and 4 mM gallic acid (**d**). In panels a to d, 100% relative intensity represents 1.3 x 10^4^ arbitrary units (a.u.). The panels from e to g show spectra of products obtained after incubation of 4 mg mL^-1^ PASC (**e**), 4 mg mL^-1^ PASC plus 4 mM gallic acid (**f**) and 4 mg mL^-1^ PASC plus 2 µM LPMO (**g**). In panels e to g, 100% relative intensity represents 1.0 x 10^4^ arbitrary units.
