# Supplementary material for: Characterisation of the enzyme transport path between shipworms and their bacterial symbionts
Source: BMC Biol. 2021 Nov 1;19:233. doi: 10.1186/s12915-021-01162-6 (PMC8561940; doi:10.1186/s12915-021-01162-6)
Supplement: Supplementary file 14 — Additional file 14: Table S6. Primers used for the cloning of the bacterial proteins LpsGH5_8, LpsGH11, LpsGH134a and LpsGH134b. LpsAA10A could not be amplified from the cDNA and therefore a synthetic version of the gene was codon-optimised for E-coli expression. File format .DOCX. [file 12915_2021_1162_MOESM14_ESM.docx]

**Additional file 14. Primers used for the cloning of the bacterial proteins *Lp*sGH5_8, *Lp*sGH11, *Lp*sGH134a and *Lp*sGH134b.** *Lp*sAA10A could not be amplified from the cDNA and therefore a synthetic version of the gene was codon-optimised for *E-coli* expression.

| ***Lp*sGH5_8** | Forward primer: GTTTCAGAGTAGTTAGCTGAACCAGCC |
| --- | --- |
|  | Reverse primer: GAGTGTCGAGTTAGCACGGTGG |
| ***Lp*sGH11** | Forward primer: ACTAGCTACCTTTCACTCAAGTTGTG |
|  | Reverse primer: CAGCCGTTGTTTTTAGTTACAGCT |
| ***Lp*sGH134a** | Forward primer: CACGGTACTCACACATACACAAATATAG |
|  | Reverse primer: ATTGGCTTAACCAGGGTAAGC |
| ***Lp*sGH134b** | Forward primer: TATGGGTGCAAACTTAAGCTGC |
|  | Reverse primer: GTATCGTTGGATTGGCTTAACG |
